# Podosome formation promotes plasma membrane invagination and integrin-β3 endocytosis on a viscous RGD-membrane

**DOI:** 10.1038/s42003-020-0843-2

**Published:** 2020-03-13

**Authors:** Fakun Cao, Yuhuan Zhou, Xiaoting Liu, Cheng-han Yu

**Affiliations:** School of Biomedical Sciences, Li Ka Shing Faculty of Medicine, University of Hong Kong, Hong Kong, China

**Keywords:** Cytoskeleton, Endocytosis, Cell adhesion

## Abstract

Integrin receptors orchestrate cell adhesion and cytoskeletal reorganization. The endocytic mechanism of integrin-β3 receptor at the podosome remains unclear. Using viscous RGD-membrane as the model system, here we show that the formation of podosome-like adhesion promotes Dab2/clathrin-mediated endocytosis of integrin-β3. Integrin-β3 and RGD ligand are endocytosed from the podosome and sorted into the endosomal compartment. Inhibitions of podosome formation and knockdowns of Dab2 and clathrin reduce RGD endocytosis. F-actin assembly at the podosome core exhibits protrusive contact towards the substrate and results in plasma membrane invaginations at the podosome ring. BIN1 specifically associates with the region of invaginated membrane and recruits DNM2. During the podosome formation, BIN1 and DNM2 synchronously enrich at the podosome ring and trigger clathrin dissociation and RGD endocytosis. Knockdowns of BIN1 and DNM2 suppress RGD endocytosis. Thus, plasma membrane invagination caused by F-actin polymerization promotes BIN1-dependent DNM2 recruitment and facilitate integrin-β3 endocytosis at the podosome.

## Abstract (web summary)

Cao et al. investigate the mechanism of integrin-β3 endocytosis on podosomes from cells on viscous RGD- membranes. By using live imaging, the authors monitor actin and membrane dynamics during podosome formation and show that integrin-β3/RGD endocytosis is DAB2/clathrin mediated and dynamin-2 and BIN1 dependent.

## Introduction

Signaling at the integrin-mediated adhesion regulates cell motility and tissue organization^1,2^. Through the binding of extracellular matrices, activated integrins can recruit adhesion adaptor proteins (e.g. talin, FAK, and paxillin) and assemble different types of cell–matrix adhesions, including focal adhesion, podosome, and invadopodium^3,4^. In particular, podosomes exhibit a short lifetime and can be found in various cell types, including macrophage, dendritic cell, smooth muscle cell, and fibroblast on viscous matrices^5,6^. Each podosome is about 0.5–2 µm in diameter and exhibits a distinct core and ring organization^7,8^. The podosome core consists of branched F-actin network polymerized by N-WASP and Arp2/3^9,10^. The podosome ring contains integrin receptors and adhesion adaptor proteins and surrounds the podosome core. Unlike contractile F-actin stress fibers attached to focal adhesions, the branched actin network of podosome core is protrusive towards the extracellular space^11^.

Disassembly of integrin-mediated adhesion is an essential process to facilitate cell migration. Abnormal integrin endocytosis has been reported to promote cell migration and cancer metastasis^12,13^. Various endocytic pathways, such as clathrin-mediated, clathrin-independent, caveolin-dependent, phosphatidylinositol-dependent endocytosis of integrin receptors have been reported^14,15^. In particular, Dab2 and Numb can directly bind to the NPxY motif of integrin β3-subunit cytoplasmic tails^16^, facilitate the assembly of clathrin coat, and promote focal adhesion disassembly^17^. Membrane curvature modulations by F-actin polymerization are also critical factors in receptor endocytosis. BIN/Amphiphysin/Rvs (BAR) domain proteins can bind to curved membrane and promote the recruitment of dynamin to the membrane fission site^18,19^. Dynamin is multidomain GTPase involved in endocytosis and remodeling of membrane organelles^20^. Upon GTP hydrolysis, dynamin oligomers collectively undergo conformational changes, resulting in the fission of endocytic pits and the release of endocytic vesicles.

Arg-Gly-Asp (RGD) peptide derived from fibronectin is one of the key ligands to activate integrin-αVβ3 heterodimer and trigger adhesion formation^21,22^. RGD supported lipid bilayer membrane (RGD-membrane) has been used as a model platform to study adhesion signaling of integrin-β3 under low traction force conditions^23,24^. In particular, viscous RGD-membrane can promote embryonic fibroblasts, which normally form contractile focal adhesions to assemble podosome-like adhesions^6,25^. Podosome-like adhesions of the embryonic fibroblast on RGD-membrane shares similar core/ring components and lifetime as macrophage podosomes. Because of their remarkable similarity, the podosome-like adhesion on RGD-membrane is referred to the podosome. While integrin endocytosis at the focal adhesion has been well studied, how activated integrin-β3 receptor undergoes endocytosis at the podosome remains unclear. Here, we report that membrane invagination resulted from protrusive F-actin polymerization triggers integrin-β3 endocytosis at the podosome.

## Results

### Dab2 localizes at integrin-β3 in the podosome ring

**Fig. 1:**
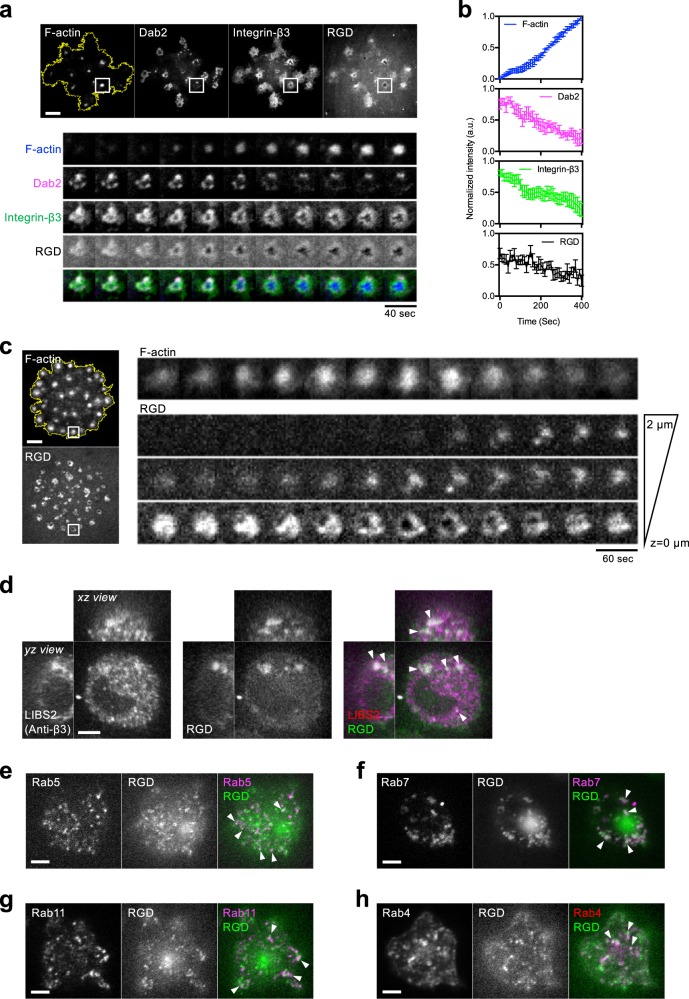
Integrin-β3 and RGD ligands are internalized at the podosome. **a** Podosome formation of REF52 cell on RGD-membrane. F-actin, marked by BFP2-UtrCH gradually polymerizes and forms the podosome core. RGD-NA680 and integrin-β3-GFP clusters reorganize into doughnut-shaped adhesion and form the podosome ring. mCherry-Dab2 localizes at integrin-β3-GFP at the podosome ring and dissociates during podosome formation. Inset: the boxed region (5 × 5 μm^2^). See Supplementary Movie 1. **b** The intensities of Dab2, integrin-β3, and RGD decrease as F-actin polymerizes. Nine podosomes from six cells in four independent experiments are analyzed. **c** RGD-NACB are internalized during podosome formation. As F-actin polymerizes (marked by GFP-UtrCH), the intensity of RGD-NACB at the adhesion plane decreases, while that at 1 and 2 µm above the adhesion plane increase. Inset: the boxed region (3 × 3 μm^2^) (see Supplementary Movie 2). **d** Internalized RGD puncta as a marker of endocytosed integrin-β3. RGD-NA488 colocalizes with activated integrin-β3 (stained by conformation-specific LIBS2 antibody) in the endosomal compartment (arrowheads). Three-dimensional confocal images are shown with the *z* position from 2 to 20 µm (*xz* and *yz* view, 500 nm *z*-step), while the image shown in *xy* view is at the *z* position of 5.5 µm above the adhesion plane. **e**–**h** After the internalization, RGD-NA488 are dynamically sorted and localize at mCherry-Rab5-positive early endosome, DsRed-Rab7-positive late endosome, tdTomato-Rab11-positive slow recycling endosome, and mCherry-Rab4-positive fast recycling endosome. The images are taken at the *z* position of 2 µm above adhesion plane (see Supplementary Figs. 2, 10b). Scale bars represent 5 μm. Error estimates are SEM.

REF52 fibroblast cells formed micron-sized RGD clusters as adhesion sites on RGD-membrane (Fig. 1a). Integrin-β3 receptors were enriched at RGD clusters and recruited cytosolic adaptor protein Dab2 (Supplementary Fig. 1a–c). Clathrin light chain (CLC) was also found in Dab2-positive integrin-β3 clusters (Supplementary Fig. 1d). As F-actin started to polymerize within the RGD–integrin-β3 cluster and formed the podosome core, solid micron-sized adhesion transformed into a doughnut-shape podosome ring (Supplementary Movie 1). Dab2 colocalized with a subpopulation of integrin-β3 at the podosome ring (Fig. 1a). Other adhesion adaptor proteins, such as talin, were also found at the podosome ring and exhibited a mutually exclusive pattern with Dab2 (Supplementary Fig. 1e, f).

### Integrin-β3 and RGD ligand are internalized at the podosome

As F-actin increasingly polymerized at the podosome core, the intensities of Dab2, integrin-β3, and RGD at the podosome ring gradually decreased (Fig. 1b). The lost RGD puncta were subsequently observed above the adhesion plane and internalized into the cell (Fig. 1c and Supplementary Movie 2). Internalized RGD puncta colocalized with the confirmation-specific antibody of activated integrin-β3 (Fig. 1d) and were dynamically sorted into Rab5, Rab7, Rab11, and Rab4 positive endosomes (Fig. 1e–h and Supplementary Fig. 2). When Dab2 and clathrin heavy chain were individually knocked down by siRNA, podosome formation were not affected (Supplementary Fig. 3a, b). However, the amount of endocytosed RGD inside the cell (RGD endocytosis level, summed intensities within 2–20 µm above the adhesion plane) became significantly reduced (Supplementary Fig. 3c–f).

### Inhibitions of podosome formation suppress RGD endocytosis

**Fig. 2:**
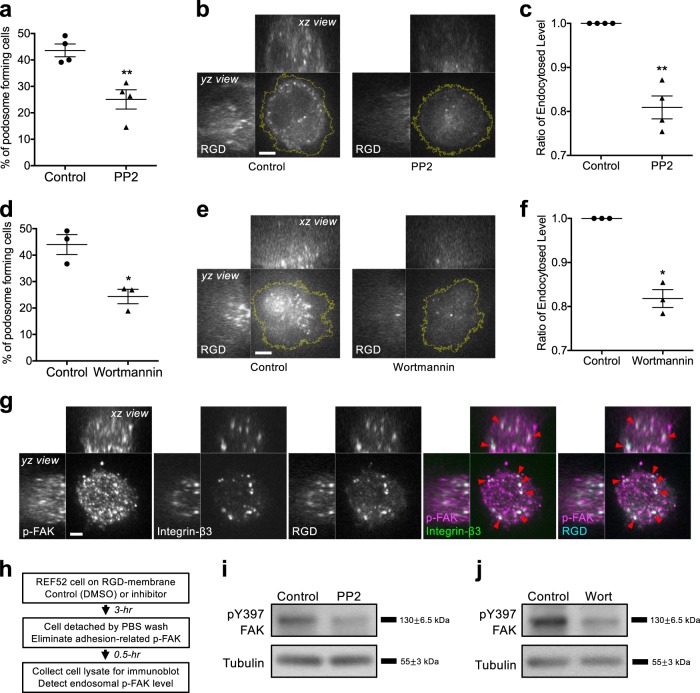
Inhibitions of podosome formation suppress RGD endocytosis. **a** Src inhibitor PP2 (10 µM) blocks podosome formation in REF52 cell. Statistical information is in Supplementary Fig. 10c. **b**, **c** Treatment of PP2 suppresses RGD-NA488 endocytosis. Three-dimensional confocal images are shown with the *z* position from 2 to 20 µm (*xz* and *yz* view, 500 nm *z*-step), while the image shown in *xy* view is at the *z* position of 5 µm above the adhesion plane. Statistical information is in Supplementary Fig. 10d. **d** PI3K inhibitor wortmannin (100 nM) blocks podosome formation in REF52 cell. Statistical information is in Supplementary Fig. 10c. **e**, **f** Treatment of wortmannin suppresses RGD-NA488 endocytosis. Three-dimensional confocal images are shown with the *z* position from 2 to 20 µm (*xz* and *yz* view, 500 nm *z*-step), while the image shown in *xy* view is at the *z* position of 5 µm above the adhesion plane. Statistical information is in Supplementary Fig. 10d. **g** pY397-FAK staining in the endosomal compartment colocalizes with internalized puncta of hs-integrin-β3-EGFP and RGD-NACB (arrowheads). Three-dimensional confocal images are shown with the *z* position from 1 to 15 µm (*xz* and *yz* view, 500 nm *z*-step), while the image shown in *xy* view is at the *z* position of 2 µm above the adhesion plane. **h** Schematic diagram of endosomal pY397-FAK detection. In order to eliminate the contribution of pY397-FAK from the adhesion site, cells are detached from RGD-membrane before lysis. **i**, **j** pY397-FAK levels contributed from the endosomal compartment are reduced when PP2 and wortmannin are introduced. Scale bars represent 5 μm. Error estimates are SEM.

Src and PI3K inhibition (PP2 10 µM and wortmannin 100 nM, respectively) can suppress podosome formation by impeding the activation of actin nucleators^9,26^ and resulted in the decrease of RGD endocytosis level (Fig. 2a–f and Supplementary Fig. 4). Autophosphorylation of FAK at Y397 (pY397-FAK) were found at both integrin-β3/RGD puncta in the endosomal compartment and adhesion sites on RGD-membrane (Fig. 2g and Supplementary Fig. 5a, b). The level of pY397-FAK in the endosomal compartment can be used as a surrogate to biochemically detect the level of RGD endocytosis. In order to exclusively detect the level of pY397-FAK in the endosomal compartment, the contribution of pY397-FAK signal from the adhesion site was quenched by detaching the cell from RGD-membrane and suspending in serum-free PBS for 30 min before lysis (Fig. 2h). Without cell detachment, Src and PI3K inhibition did not alter the combined pY397-FAK signal (Supplementary Fig. 5c). However, pY397-FAK levels in the endosomal compartment were reduced when cells were under the condition of Src and PI3K inhibition followed by detachment (Fig. 2i, j).

### Dynamin2 (DNM2) recruitment to the podosome ring triggers RGD endocytosis

**Fig. 3:**
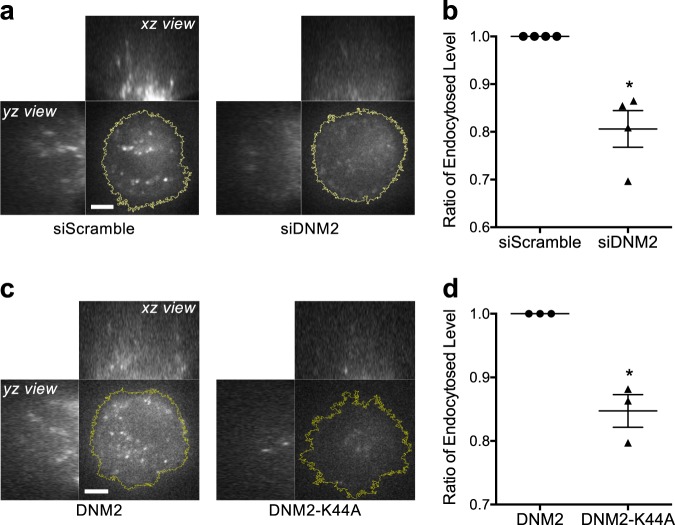
DNM2 regulates in RGD endocytosis. **a**, **b** Knockdown of DNM2 results in the decrease of RGD-NA594 endocytosis level in REF52 cell. Three-dimensional confocal images are shown with the *z* position from 2 to 20 µm (*xz* and *yz* view, 500 nm *z*-step), while the image shown in *xy* view is at the *z* position of 5 µm above the adhesion plane. Statistical information is in Supplementary Fig. 10e. **c**, **d** Introduction of dominant-negative DNM2-K44A mutant results in the decrease of RGD-NA594 endocytosis level in REF52 cell. Three-dimensional confocal images are shown with the *z* position from 2 to 20 µm (*xz* and *yz* view, 500 nm *z*-step), while the image shown in *xy* view is at the *z* position of 5 µm above the adhesion plane. Statistical information is in Supplementary Fig. 10f. Scale bars represent 5 µm. Error estimates are SEM.

**Fig. 4:**
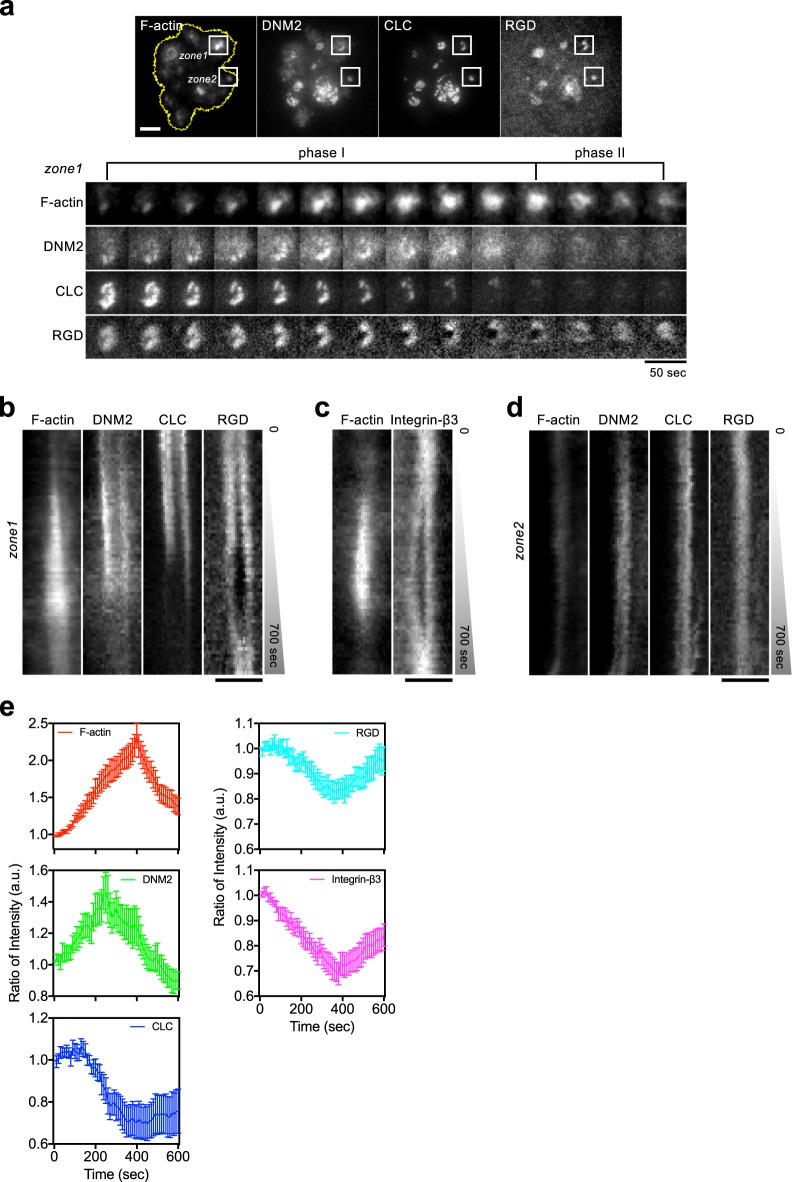
Spatiotemporal enrichment of DNM2 triggers RGD endocytosis at the podosome ring. **a** During the progressive F-actin polymerization at the podosome (phase I), DNM2-mCherry is gradually enriched at the podosome ring, while mTagBFP2-CLC and RGD-NA680 begin to dissociate. F-actin is labeled by GFP-UtrCH in REF52 cell. Inset: zone 1, podosome adhesion; zone 2, non-podosome adhesion. Insets represent 4 × 4 μm^2^ (see Supplementary Movie 3). **b** Kymograph of zone 1 in **a**. As F-actin polymerization takes place, the level of DNM2 at the podosome ring increases, and the level of CLC and RGD decreases. **c** Kymograph of F-actin and integrin-β3-BFP2 in the podosome adhesion (see Supplementary Fig. 6f). **d** Kymograph of zone2 in **a**. Without prominent F-actin polymerization, the intensities of DNM2, CLC, and RGD remain relatively unchanged. **e** Intensity analyses of F-actin, DNM2, CLC, RGD and integrin-β3 at the podosome ring. All intensity traces are synchronized by realigning F-actin peak intensity at 400 s. DNM2 reaches the peak intensity before F-actin, while the intensities of CLC, integrin-β3, and RGD progressively decreases. DNM2, CLC, and RGD intensities are analyzed from nine podosomes in five cells in three independent experiments. Integrin-β3 intensities are from 10 podosomes in five cells in three independent experiments. F-actin intensities are analyzed from the 19 podosomes mentioned above. Scale bars represent 5 µm **a** and 3 μm **b**–**d**. Error estimates are SEM.

DNM2 is one of the key regulators to pinch off clathrin-coated endocytic structures from the plasma membrane. Knockdown of DNM2 and overexpression of dominant negative mutant DNM2-K44A resulted in the decrease of RGD endocytosis level without interfering the podosome formation (Fig. 3 and Supplementary Fig. 6a–e). During the progressive F-actin polymerization at the podosome (phase I), there was a distinct increase of DNM2 recruitment to the podosome ring while CLC began to dissociate (zone 1 in Fig. 4a and Supplementary Movie 3). DNM2 reached its peak intensity before F-actin, and CLC became completely dissociated from the podosome ring (Fig. 4b, e). At the same time, the intensities of RGD and integrin-β3 at the podosome ring gradually decreased (Fig. 4b, c, e, and Supplementary Fig. 6f). When F-actin started to depolymerize (phase II), DNM2 remained absent, and the intensities of RGD and integrin-β3 gradually increased. Without prominent F-actin assembly and podosome formation, the intensities of RGD, CLC and DNM2 remained relatively unchanged (zone 2 in Fig. 4a, d).

### Protrusive F-actin polymerization causes plasma membrane deformation at the podosome ring

**Fig. 5:**
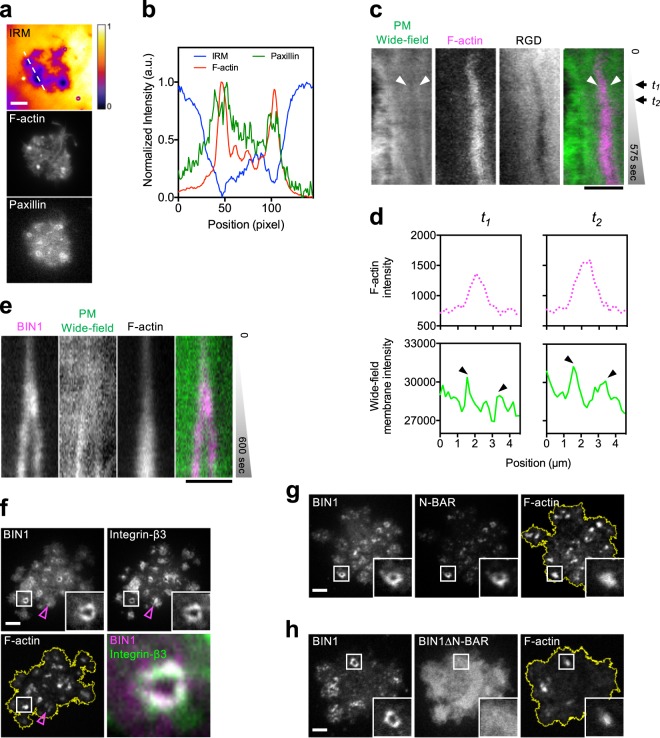
Protrusive F-actin polymerization results in plasma membrane invagination and BIN1 recruitment to the podosome ring. **a** Interference reflection microscopy (IRM) image of REF52 cell on RGD-membrane. **b** Intensity profiles of IRM, F-actin, and YFP-paxillin along the dashed line in **a**. The region of podosome core (F-actin, labeled by CF594-phalloidin) exhibits the lowest intensity in the IRM channel and represents the close contact and protrusive F-actin polymerization towards the substrate. **c** Kymograph of plasma membrane (PM, labeled by mCherry-KRas-CT), F-actin (labeled by BFP2-UtrCH), and RGD-NA680 during the podosome formation in MEF cell. Plasma membrane becomes enriched and encircles the protrusive F-actin (arrowheads) (see Supplementary Fig. 7c). **d** Intensity profiles at *t*_1_ and *t*_2_ in **d**. The intensities of plasma membrane gradually increase around the F-actin (arrowheads). **e** Kymograph of BIN1-mCherry, plasma membrane (PM, labeled by PM-GFP), and F-actin (labeled by BFP2-UtrCH) during the podosome formation in MEF cell. BIN1 localizes at the site of plasma membrane invagination and encircles the protrusive F-actin (see Supplementary Fig. 7d and Supplementary Movie 4). **f** BIN1-mCherry specifically colocalizes with integrin-β3-GFP at the podosome ring and surrounds dot-like F-actin assembly of the podosome core. F-actin is labeled by BPF2-UtrCH in REF52 cell. Inset: the boxed region (5 × 5 μm^2^). **g**, **h** N-BAR-GFP (aa 1–267), not BIN1∆N-BAR-GFP (aa 268–476) colocalizes with BIN1-mCherry around BFP2-UtrCH labeled podosome core in REF52 cell. Inset: the boxed region (5 × 5 μm^2^). Scale bars represent 5 µm.

While the underlying supported lipid membrane remained intact, the RGD intensity at the podosome core was lower than the background intensity of RGD-membrane (Supplementary Fig. 7a, b). The local depletion of RGD implied the involvement of physical perturbations that counteracted the diffusive characteristics of supported lipid membrane. By interference reflection microscopy (IRM), the region of podosome core exhibited the lowest intensity, indicating the close contact and protrusive F-actin polymerization towards the supported lipid membrane (Fig. 5a, b). As F-actin progressively polymerized at the podosome core, the intensity of the plasma membrane, visualized by membrane marker mCherry-KRas-CT became enriched around the F-actin (arrowheads in Fig. 5c and Supplementary Fig. 7c). Intensity profiles by line scan indicated two distinct peaks of mCherry-KRas-CT surrounding a single peak of F-actin (arrowheads in Fig. 5d). The intensity rises of the plasma membrane around the protrusive podosome core suggested membrane deformation or stacking of multiple lipid bilayer structures at the podosome ring.

### BIN1 is recruited to the deformed plasma membrane at the podosome ring

BIN1, a curvature-sensing N-BAR domain protein was associated with the deformed plasma membrane around protrusive F-actin assembly and colocalized with integrin-β3 at the podosome ring (Fig. 5e, f, Supplementary Fig. 7d, and Supplementary Movie 4). The recruitment of BIN1 was specific to podosome adhesions, as BIN1 was absent at non-podosome adhesions that lacked protrusive F-actin polymerization (empty arrowhead in Fig. 5f). N-BAR domain of BIN1 (aa 1–267) colocalized with full length BIN1 at the podosome ring (Fig. 5g). BIN1 without N-BAR domain (BIN1∆N-BAR, aa 268–476) became cytosolic and was not enriched at the podosome ring (Fig. 5h). Other SH3-containing BAR-domain proteins, including FBP17 and syndapin2 did not distinctly enrich at the podosome (Supplementary Fig. 8a, b). CIP4, a F-BAR domain containing protein was found at the podosome core but did not localized at the plasma membrane enrichment around the polymerizing F-actin (Supplementary Fig. 8c)

### BIN1 recruits DNM2 to the podosome ring and promotes RGD endocytosis

**Fig. 6:**
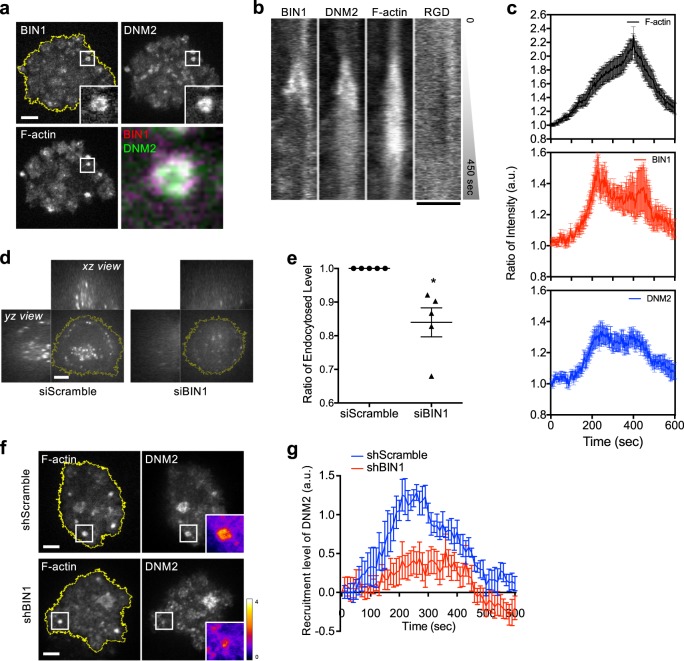
BIN1 recruits DNM2 to the podosome ring and promotes RGD endocytosis. **a** BIN1-mCherry colocalizes with DNM2-GFP at the podosome ring. F-actin is labeled by BPF2-UtrCH in REF52 cell. Inset: the boxed region (4 × 4 μm^2^) (see Supplementary Movie 5). **b** Kymograph of the boxed region of **a**. BIN1 and DNM2 are enriched at the podosome ring in the early phase of podosome formation. **c** Intensity analyses of BIN1 and DNM2 at the podosome ring. All intensity traces are synchronized by realigning F-actin peak intensity at 400 s. BIN1 and DNM2 both reach the peak intensity before F-actin. Eighteen podosomes from nine cells in three independent experiments are analyzed. **d**, **e** Knockdown of BIN1 suppresses RGD-NA488 endocytosis. Three-dimensional confocal images are shown with the *z* position from 2 to 20 µm (*xz* and *yz* view, 500 nm *z*-step), while the image shown in *xy* view is at the *z* position of 5 µm above the adhesion plane. Statistical information is in Supplementary Fig. 10g. **f** Knockdown of BIN1 by shRNA suppresses the recruitment of DNM2-GFP to the podosome ring. Inset: the boxed region (5 × 5 μm^2^). Ratiometric insets of DNM2 indicate the recruitment level (see Supplementary Movie 6). **g** Recruitment level of DNM2-GFP during podosome formation. The recruitment of DNM2 to the podosome ring is reduced when BIN1 expression is suppressed. All intensity traces are synchronized by realigning F-actin peak intensity at 400 s. shScramble samples are analyzed from nine podosomes from six cells in four independent experiments. shBIN1 samples are analyzed from 12 podosomes from seven cells in five independent experiments. Scale bars represent 5 µm. Error estimates are SEM.

During the podosome formation, BIN1 and DNM2 exhibited similar spatiotemporal recruitments around the F-actin core and colocalized at the podosome ring (Fig. 6a, b, Supplementary Fig. 10a, and Supplementary Movie 5). Like DNM2, BIN1 also reached the peak intensity in the early phase of podosome formation before F-actin (Fig. 6c). Knockdown of BIN1 caused the decrease of RGD endocytosis level without interfering podosome formation (Fig. 6d–f and Supplementary Fig. 9a, b). Knockdown of CIP4, on the other hand, did not suppress RGD endocytosis (Supplementary Fig. 8d–e). It is known that C-terminal SH3 domain of BIN1 can directly interact with DNM2’s PRD domain^27^ (Supplementary Fig. 9c). BIN1∆SH3 (aa 1–404) was found at the podosome ring while DNM2∆PRD (aa 1–742) was diffusive within the cell and did not enriched at the podosome (Supplementary Fig. 9d, e). When BIN1 was knocked down, DNM2 recruitment to the podosome ring was significantly reduced (Fig. 6f, g and Supplementary Movie 6). Overexpression of BIN1∆SH3 also impeded the recruitment of DNM2 to the podosome ring (Supplementary Fig. 9d, h) and resulted in the reduction of RGD endocytosis level (Supplementary Fig. 9f–g).

## Discussion

Podosomes are integrin-mediated adhesions, and each podosome consists of the unique ring and core organizations. On RGD-membrane, integrin-β3 adhesion binds to RGD ligand and forms the adhesion ring of the podosome. Here, we find that endocytic adaptor protein Dab2 and clathrin localize at the podosome ring and subsequently dissociate as F-actin progressively polymerizes at the podosome core. Concurrently, integrin-β3 and RGD ligand at the podosome ring gradually diminish and are internalized. Puncta of RGD ligand colocalize with activated integrin-β3 and pY397-FAK at the endosomal compartment and therefore are used as a surrogate to measure the endocytosis level of integrin-β3. While RGD puncta are found in early endosomes (Rab5-positive), late endosomes (Rab7-positive), slow-recycling endosomes (Rab11-positive), and fast-recycling endosomes (Rab4-positive), higher degrees of RGD enrichment at Rab7 and Rab4-positive endosomes are observed. Blockages of actin nucleator activation by Src and PI3K inhibition suppress podosome formation and cause the decrease of the amount of endocytosed RGD and endosomal pY397-FAK level.

Protrusive F-actin polymerization is visualized by IRM and results in the invagination of the surrounding plasma membrane. Close association with the underlying supported bilayer substrate accounts for the physical perturbation that causes the local depletion of RGD intensity at the podosome core. The concave profile of the deformed plasma membrane around the podosome core is demonstrated by gradual enrichment of plasma membrane markers and the specific recruitment of BAR-domain protein that recognizes and binds to lipid membrane with high curvature^28,29^. In particular, BIN1, a N-BAR domain protein, is specifically recruited around the podosome core and is absent from non-podosome adhesions. BIN1 localizes at the membrane invagination via its N-BAR domain and recruits membrane fission regulator DNM2 to the podosome ring via its C-terminal SH3 domain. Knockdowns of Dab2, clathrin heavy chain, BIN1, and DNM2 and overexpression of BIN∆SH3 and DNM2-K44A mutants result in the decrease of RGD endocytosis level. CIP4, a F-BAR containing protein is enriched at the podosome core and is also known to interact with DNM2 via its SH3 domain^30,31^. Intriguingly, knockdown of CIP4 does not cause a significant decrease of RGD endocytosis level.

**Fig. 7:**
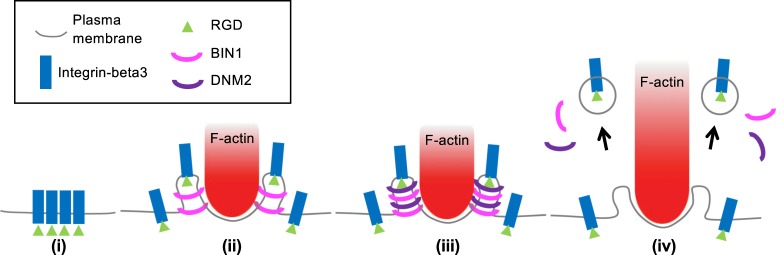
Working model of integrin-β3 endocytosis at the podosome. (i) Integrin-β3 form clusters on RGD-membrane. (ii) Podosome formation. Protrusive F-actin polymerization leads to plasma membrane invagination. BIN1 binds the curved membrane at the podosome ring. (iii) BIN1 recruits DNM2 to the membrane invagination site. (iv) DNM2 triggers membrane scission and RGD–integrin-β3 endocytosis.

Spatiotemporal enrichment of BIN1 and DNM2 at the podosome ring is the key event to trigger the endocytosis of integrin-β3. Knockdown of BIN1 and overexpression of BIN1∆SH3 mutant result in the poor recruitment of DNM2. It appears that BIN1 serves as an upstream regulator to recruit DNM2 to the invaginated membrane at the podosome ring. Both BIN1 and DNM2 progressively enrich at the podosome ring in the early phase of podosome formation and reach the peak intensity earlier than F-actin. At the same time, the intensities of integrin-β3 and RGD ligands start to diminish. Podosome formation and receptor-mediated endocytosis share many similar molecular events, including endocytic adaptor binding, membrane curvature modulation, Arp2/3-mediated F-actin polymerization, and dynamin-mediated fission^32,33^. In the case of membrane receptors with soluble ligands, the assembly of clathrin triskelion is the main driving force for plasma membrane bending, and BAR domain proteins sense and stabilize the membrane curvature. However, as integrin-β3 receptors are physically anchored to extracellular RGD ligands, membrane bending around ligand-bound integrin-β3 receptors solely by the assembly of clathrin triskelion may not be energetically favorable. Protrusive F-actin polymerization is necessary to trigger plasma membrane invagination and BIN1 accumulation around the podosome core. BIN1-mediated DNM2 recruitment then triggers the fission of invaginated membrane and promotes the endocytosis of integrin-β3 at the podosome ring (Fig. 7).

Integrin-mediated adhesions can activate ERK and PI3K signaling via autophosphorylated FAK and support cell growth^34^. On the other hand, integrin endocytosis can promote anchorage-independent cell survival of cancer cells^35^. Viscous RGD-membrane may resemble the microenvironment of cleaved extracellular matrices by matrix metalloproteinases of invasive cancer cells^36^. Matrix-bound integrin receptors can remain in the active conformation after endocytosed and recruit FAK to the endosome. Without cell attachment to matrices, autophosphorylation of pY397-FAK contributed from active integrin receptors in the endosome can continue to support ERK and PI3K signaling^37,38^. Likewise, integrin endocytosis and recycling play an important role in cell migration. While each podosome adhesion can turn over within 2–12 min^7^, protrusive characteristics of the podosome can locally promote the plasma membrane invagination and trigger the disassembly of podosome via integrin receptor endocytosis. In this study, we demonstrate a podosome-specific and membrane curvature-dependent pathway to facilitate integrin-β3 endocytosis. It remains to be investigated whether endocytosed integrin-β3 recycles to the plasma membrane to promote new adhesion formation and cell migration.

## Methods

### Preparation of RGD-membrane

1,2-dioleoyl-sn-glycero-3-phosphocholine (DOPC) and 1,2-dioleoyl-sn-glycero-3-phosphoethanolamine-N-(cap biotinyl) (biotinyl-Cap-PE) were purchased from Avanti Polar Lipids (850375C and 870273C, respectively). Lipids with desired compositions were mixed in chloroform and dried by rotatory evaporation in 50–60 °C water bath. The lipids were then rehydrated with 2 ml Milli-Q water in 4 °C fridge overnight. To make small unilamellar vesicles (SUVs), the lipids suspension was probe-sonicated in an ice bath and then centrifuged at 16,000×*g* for 3 h at 4 °C. Supernatant solution of SUVs was collected and stored at 4 °C. Glass substrate (25 mm in diameter, #1.5) was prepared by bath-sonication for 30 min, and then was immersed in freshly prepared 50% sulfuric acid overnight, rinsed with 50 ml Milli-Q water 10 times, and finally dried under a nitrogen gas stream. Supported lipid membrane was self-assembled by incubating the mixture of lipid SUVs (99.8 mol% of DOPC and 0.2 mol% of biotinyl-Cap-PE) and an equal volume of phosphate-buffered saline (PBS) on the glass substrate in room temperature^22^. Excess lipid SUVs were removed in a Milli-Q water bath. The glass substrate with supported lipid membrane was assembled with an Attofluor Cell chamber (Thermo Fisher Scientific A7816) and was always kept under the aqueous condition. Membrane detects were blocked by a 30-min incubation of BSA in PBS buffer (1 mg/mL). 1.5 µg of neutravidin (NA, Thermo Fisher Scientific) with no tag (A2666), Cascade Blue (NACB, A2663), DyLight 488 (NA488, 22832), DyLight 594 (NA594, 22842), or DyLight 680 (NA680, 22848) was then introduced onto the supported lipid membrane for 30 min and then washed away by 25 ml of PBS buffer. Subsequently, 1.5 µg of cyclo [Arg-Gly-Asp-D-Phe-Lys(Biotin-PEG-PEG)] (RGD, PCI-3697-PI, Peptides International) was introduced onto neutravidin-coated supported lipid membrane for 30 min and then washed away by 25 ml of PBS buffer. Before seeding cells, each chamber with RGD-membrane was rinsed with 15 ml serum-free DMEM medium and kept in 37 °C. Unless otherwise stated, live cell imaging began 90 min after seeding cells on RGD-membrane.

### Cell culture

Rat embryonic fibroblast (REF52) and REF52 stably expressing YFP-paxillin were gifts from Dr. Benjamin Geiger, Weizmann Institute of Science, Rehovot, Israel. RPTPα^+/+^ mouse embryonic fibroblast (MEF) was a gift from Dr. Sap Jan, New York University School of Medicine^39^. REF52 and MEF cell lines were cultured in DMEM medium (Sigma D1152) supplemented with 10% (v/v) fetal bovine serum (Hyclone SV30160.03), 100 U/ml penicillin–streptomycin (Thermo Fisher Scientific 15140122) in 37 °C incubator with 5% CO_2_. MEF cell was used in the membrane invagination experiment, as REF52 cell exhibited various membrane ruffling that often resulted in high fluctuation and uneven background signal of the plasma membrane. Unless otherwise stated, REF52 cells were utilized in all other experiments.

### Plasmid and siRNA/shRNA

Plasmid and siRNA/shRNA were transiently transfected via Neon electroporation system (Thermo Fisher Scientific). For plasmids, experiments were performed 24–48 h after electroporation. For siRNA and shRNA, experiments were performed 72 h after electroporation. Plasmids include Integrin-β3-GFP^40^, mCherry-Dab2^41^, GFP-Dab2^41^, GFP-UtrCH (Addgene 26737), mch-Rab5 (Addgene 49201), DsRed-rab7 WT (Addgene 12661), tdTomato-Rab11a-7 (Addgene 58128) DNM2-pmCherryN1 (Addgene 27689), wt DNM2 pEGFP (Addgene 34686), K44A DNM2 pEGFP (Addgene 34687), CLC-pmCherryC1 (Addgene 27680), PM-GFP (Addgene 21213), BIN1-pmCherryN1 (Addgene 27693), CIP4-pmCherryC1 (Addgene 27685), FBP17-pmCherryC1 (Addgene 27688), and Syndapin2-pmCherryC1 (Addgene 27681). Talin-GFP, FAK-EGFP, Integrin-β3-BFP2, and BPF2-UtrCH were used in our previous work^6,23^. mTag-BFP2 CLC, mCherry-Rab4a, human integrin-β3 was a gift from Pakorn Kanchanawong, Mechanobiology Institute, National University of Singapore. Human integrin-β3-EGFP (hs-integrin-β3-EGFP) was generated by PCR and subcloned into pEGFP-N1 vector. N-BAR domain of BIN1 (aa 1–267) and BIN1∆N-BAR (aa 268–476) were generated by PCR of BIN1-pmCherryN1 and subcloned into pEGFP-N1 and pmCherryN1 vector. BIN1∆SH3 (aa 1–404) was generated by PCR of BIN1-pmCherryN1 and subcloned into mRuby2-N1 vector (Addgene 54614). DNM2∆PRD (aa1–742) was generated by PCR of DNM2-pmCherryN1 and subcloned into pEGFP-N1 vector. siGLO Red transfection indicator (D-001630-02-05) was purchased from Dharmacon. Scramble and siRNAs targeted Rat for BIN1(SR511599), DNM2 (SR501378), Dab2 (SR505444), Clathrin heavy chain (Cltc) (SR507164), CIP4 (SR514456), and pRFP-C-RS vector used in shBIN1 were obtained from Origene (Supplementary Table 1).

### Inhibition chemicals

PP2 and wortmannin were purchased from Selleckchem. Chemicals were first kept as a stock concentration 1000 times higher than the final concentration. Before applying to cells, chemicals were diluted 1000 times into DMEM media.

### Fluorescence microscopy

Fluorescent images were taken by an inverted spinning-disc confocal microscope (Perkin-Elmer Ultraview VoX, Yokogawa CSU-X1, Nikon Eclipse Ti-E) and an inverted total internal reflection fluorescence (TIRF) microscope (iLas2, Roper Scientific, Zeiss Axio Observer Z1). An EMCCD camera (Hamamatsu C9100-23B), ×100 oil immersion lens (NA = 1.45), AOTF-controlled solid-state lasers (40–50 mW), and a piezo Z stage were equipped on the spinning-disk confocal microscope, and Volocity software (Perkin-Elmer) was used to control image acquisition. TIRF microscope contained an EMCCD camera (Photometrics Evolve 512), ×100 oil immersion lens (NA = 1.46), and AOTF-controlled solid-state lasers (50–100 mW), and was controlled by image acquisition software MetaMorph (Molecular Devices). Structured illumination super-resolution images were taken by Zeiss ELYRA S1 (SR-SIM) with ×63 oil immersion lens (1.40 NA) and cooled PCO Edge sCMOS camera. AOTF-controlled solid-state lasers (50–150 mW) were mounted on the microscope body (Zeiss Axio Observer Z1), and ZEN 2.1 software (Zeiss) was used to control image acquisition. A widefield epifluorescence microscope (Nikon Eclipse Ti-E) equipped with an IRM filter cube (50/50 beam splitter as dichroic mirror and 530/11 nm excitation filter), a high-pressure mercury lamp, and ×100 oil immersion lens (NA = 1.45) was used to perform IRM. An environmental chamber (37 °C and 5% CO_2_) was attached to the microscope body for long-term time-lapse imaging. Phenol red-free DMEM (Thermo Fisher Scientific 11054-020) with 20 mM HEPES (Thermo Fisher Scientific 15630-080) was used as the imaging medium.

### Quantification of podosome-forming cells

The cell was identified as “podosome-forming cell” when one podosome was found. Podosome was defined as the RGD ring assembly and a dense F-actin core. In addition, RGD intensity at the F-actin core had to be depleted and be lower than the background intensity of RGD-membrane (see Supplementary Fig. 7a, b).

### Quantification of RGD endocytosis level

After 3-h adherence on RGD-membrane, cells were treated with 4% paraformaldehyde at 37 °C for 20 min first and then 0.1% Triton X-100 treatment at 37 °C for 20 min. Under identical image acquisition parameters, fixed cells with endocytosed RGD were imaged by a spinning disc confocal microscopy with a z-step of 500 nm. Z-stack images from 2 to 20 µm were summed together, and the region of interest of the entire cell (ROI) was defined by ImageJ software. Intensity within the cell in the summed image (*I*_total_), camera background intensity (*I*_BKG_), and area of ROI (*A*_ROI_) were measured accordingly. The level of RGD endocytosis was then defined as (*I*_total_−*I*_BKG_) × *A*_ROI_. The ratio of endocytosis level was calculated by Excel software and plotted by GraphPad Prism software.

### Bleach correction

RGD aggregates outside the cell were used as the bleached correction standard during the time-lapsed imaging acquisition. Intensities were measured by the “Spot function” of Imaris software (Bitplane). An intensity correction curve was then generated, and bleach-corrected RGD intensity was defined by multiplying the correction curve to the raw intensity value. For time-lapsed images, kymographs, and supplementary movies, bleach corrections of fusion proteins were performed by “Bleach correction by simple ratio” in ImageJ software. Images were uniformly and unbiasedly processed to enhance the contrast after bleach correction.

### Intensity analysis and kymograph

Intensity measurement of podosome components and drift correction were achieved by the “Spot function” of Imaris software (Bitplane). Kymograph, ROI selection, and mean intensity measurement were performed by ImageJ software. To synchronize the event, the timepoint with the highest F-actin intensity in each dataset was set to be 400-s, and the other channels was realigned accordingly. The ratio of intensity was calculated by dividing the intensity value with that of the first timepoint in each channel and was plotted by GraphPad Prism software.

### Recruitment level and ratiometric analysis of DNM2

The intensity of DNM2 at the podosome ring (*I*_t_), cellular background (*I*_C_), and camera background (*I*_BKG_) were measured by ImageJ software. The intensity of DNM2 at first time point was define as *I*_0_. Recruitment level of DNM2 over time was then defined as *(I*_t_*−I*_0_*)*/(*I*_C_*−I*_BKG_) and plotted by GraphPad Prism software. Ratiometric images were prepared by subtracting camera background and dividing cellular background in ImageJ. The identical dynamic range of ratiometric images was applied.

### Colocalization analysis

Colocalization analysis was achieved by Coloc function of Imaris software (Bitplane). Integrin-β3 was used as the reference channel. The amount of voxels (*V*) of integrin-β3 and Dab2 were measured accordingly. Voxels were identified at the adhesion plane only. The percentage of colocalization was defined by *V*_Dab2_/*V*_integrin-β3_.

### Immunofluorescent staining

After 3-h adherence on RGD-membrane, cells were treated firstly with 4% paraformaldehyde and then 0.1% Triton X-100 treatment for 20 min each at 37 °C. 5% BSA with 10% normal donkey serum (blocking buffer) was used for overnight blocking at 4 °C. Samples were then incubated with the primary antibody in blocking buffer at 4 °C for 48 h. Primary antibody was then washed with PBS, followed by secondary antibody incubation for 2 h in room temperature. Primary antibodies included activated human integrin-β3 antibody (1:25, anti-LIBS2 epitope clone ab62, Millipore MABT27) and pFAK-Y397 (1:50, Thermo Fisher Scientific 44-624G). Secondary antibodies included AF594-anti-mouse (1:1000, Thermo Fisher Scientific A-21203) and AF594-anti-rabbit (1:1000, Thermo Fisher Scientific A-21207).

### Western blotting

Cells were lysed in RIPA buffer (Thermo Fisher Scientific 89900) with protease and phosphatase inhibitors (Thermo Fisher Scientific A32959) and were boiled in 4X Laemmli buffer. Lysates were separated by SDS–PAGE on 12% gel (Bio-Rad 161-0175). Protein concentrations of the lysates were determined by Bradford assay (Bio-Rad 500-0006). Gels were transferred to PVDF membrane (Millipore IPVH00005) and blocked by 5% non-fat milk (for non-phosphorylated targets) or 5% BSA (for phosphorylated targets) for 1 h in room temperature. PVDF membranes were then incubated with 5% BSA containing primary antibody at 4 °C overnight, washed with 1X TBST, followed by secondary antibody for 1 h in room temperature. Western blots were developed by ECL (Thermo Fisher Scientific SG251207) according to the manufacturer’s instruction. Primary antibodies included pFAK-Y397 (1:1000, Thermo Fisher Scientific 44-624G), anti-BIN1 clone 99D (1:1500, Millipore 05-449), anti-DNM2 clone 27 (1:1000, BD Biosciences 610263), anti-Dab2 (D7O9T) (1:1000, Cell Signaling Technology 12906), anti-Clathrin heavy chain clone 23 (1:1000, BD Biosciences 610499), anti-CIP4 clone 21 (1:1000, BD Biosciences 612557), anti-tubulin alpha (G436) (1:10,000, Bioworld BS1699), and anti-GAPDH (1:10,000, Thermo Fisher Scientific AM4300). Secondary antibodies included anti-mouse HRP (1:2000, Santa Cruz sc-516102) and anti-rabbit HRP (1:2000, Cell Signalling Technology 7074). Unprocessed full blots can be found in Supplementary Fig. 11.

### Statistics and reproducibility

Statistical bar graphs with the mean and error bars (SEM) and significance analysis (unpaired *t* test with Welch’s correction) were prepared by GraphPad Prism software. *P* values were calculated (**P < 0.001, *P < 0.05). Detailed statistical information can be found in Supplementary Fig. 10. Each dataset contains at least three independent biological repeats.

### Reporting summary

Further information on research design is available in the Nature Research Reporting Summary linked to this article.

## Supplementary information

Supplementary Information

Description of Additional Supplementary Files

Supplementary Movie 1

Supplementary Movie 2

Supplementary Movie 3

Supplementary Movie 4

Supplementary Movie 5

Supplementary Movie 6

Supplementary Data 1 (source data)

Reporting Summary

## Data Availability

Reagents used during the study are available from the corresponding author upon request. All data supporting the findings are available within the paper and its supplementary information. Source data can be found in Supplementary Data 1.
