# Supplementary material for: Podosome formation promotes plasma membrane invagination and integrin-β3 endocytosis on a viscous RGD-membrane
Source: Commun Biol. 2020 Mar 13;3:117. doi: 10.1038/s42003-020-0843-2 (PMC7070051; doi:10.1038/s42003-020-0843-2)
Supplement: Supplementary file 1 — Supplementary Information [file 42003_2020_843_MOESM1_ESM.pdf]

SUPPLEMENTARY INFORMATION

Cao et al., Podosome formation promotes plasma membrane invagination and integrin-β3 endocytosis on a viscous RGD-membrane.

Supplementary Figure 1 to 11

Supplementary Table 1

Supplementary Figure 1

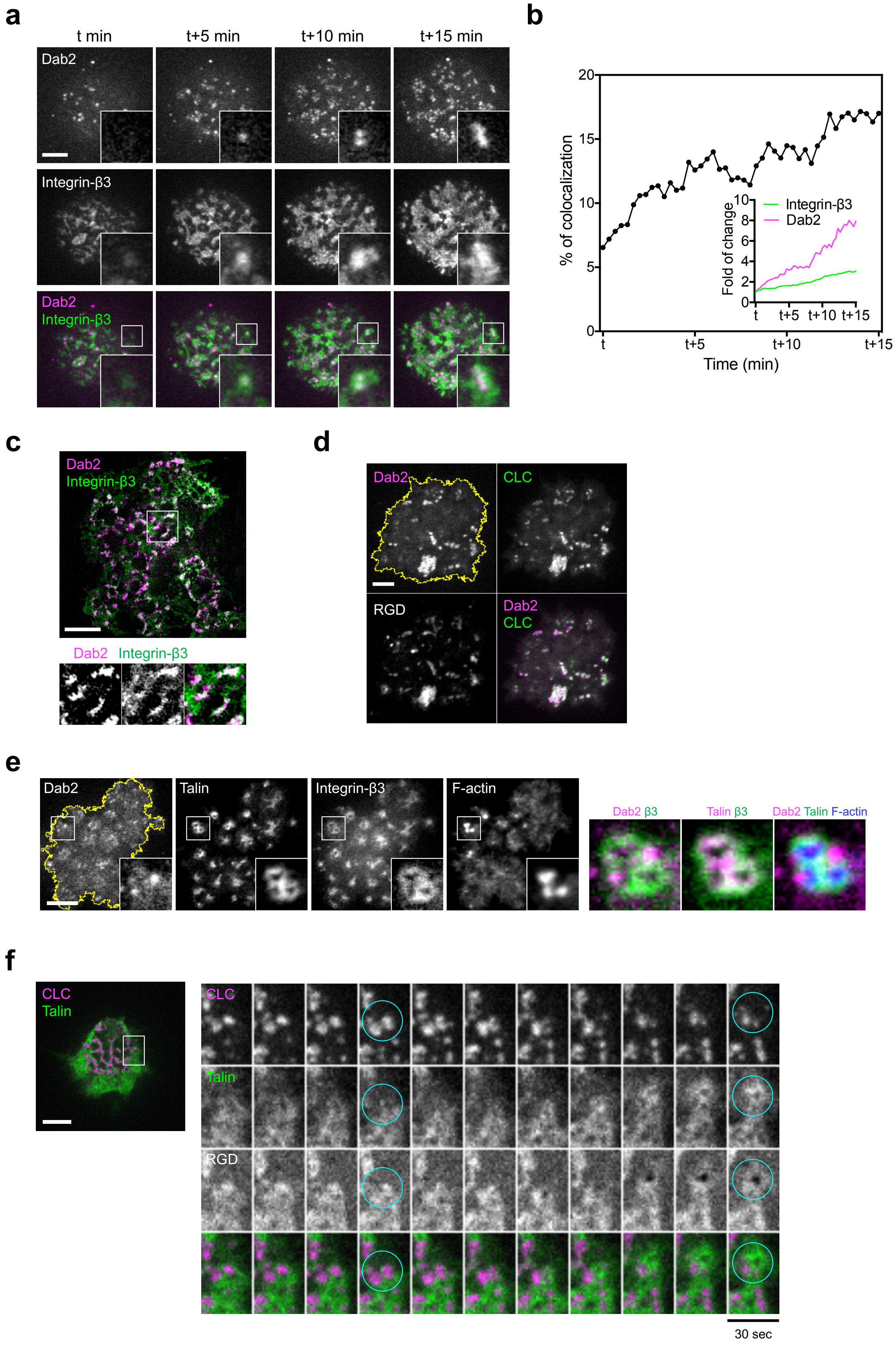

Supplementary Figure 1. (A) Integrin-β3-GFP forms micron-sized clusters when REF52

cell spreads on RGD-membrane. mCherry-Dab2 is gradually recruited to integrin-β3-GFP. After a 10-minute, 30-minute and 60-minute adherence on RGD-membrane, the ratios of integrin-β3 colocalization with Dab2 become 1.8%, 5.7% and 10.9%, respectively. Inset: the boxed region (3.4x3.4 μm^2). (B) Voxel-based colocalization analysis of integrin-β3 and Dab2 in (A). The ratio of Dab2-postitive integrin-β3 voxels increases. Voxels are identified at the adhesion plane only. Inset: the increase (fold of change) of Dab2 and integrin-β3 voxels. The increase of Dab2 voxels is faster than that of integrin-β3 voxels. (C) Structured illumination microscopy images of mCherry-Dab2 and integrin-β3-GFP. Dab2 colocalizes with a subpopulation of integrin-β3. Inset: the boxed region (5x5 μm^2). (D) mTag-BFP2 clathrin light chain (CLC) colocalizes with mCherry-Dab2-positive RGD-NA680 clusters. (E) mCherry-Dab2 and GFP-talin are both found in the podosome ring of Integrin-β3-BFP. However, Dab2 and talin exhibit a mutually exclusive pattern. Inset: the boxed region (3.5x3.5 μm^2). (F) mCherry-CLC at the podosome ring starts to dissociate while GTP-talin increases during the podosome formation (cyan circled region). Inset: the boxed region (4x6 μm^2). Scale bars represent 5 μm.

Supplementary Figure 2

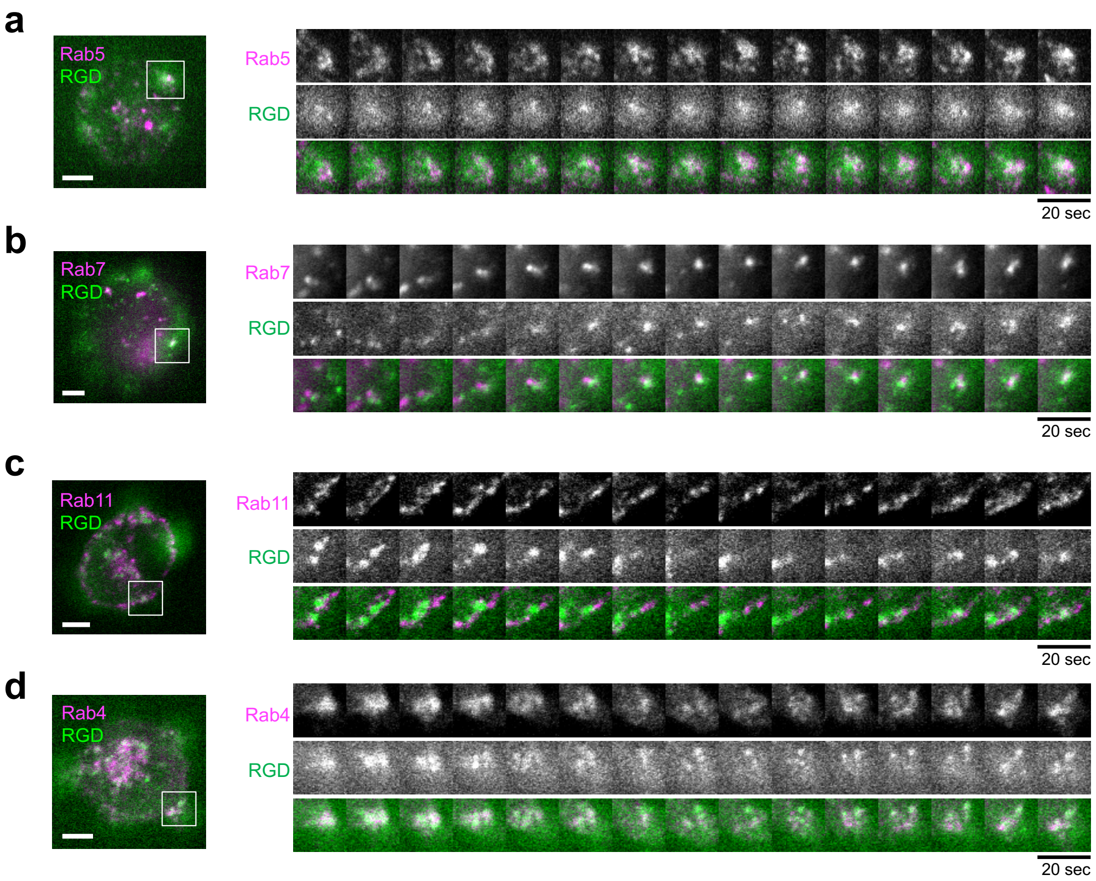

Supplementary Figure 2. Endocytosed RGD-NA488 in REF52 cell are dynamically sorted into mCherry-Rab5(A), DsRed-Rab7(B), tdTomato-Rab11(C) and mCherry-Rab4(D) positive vesicles. Time-lapse images are taken at the z position of 2μm above the RGD-membrane. Boxed region is 6x6 μm^2. Scale bars represent 5 μm.

Supplementary Figure 3

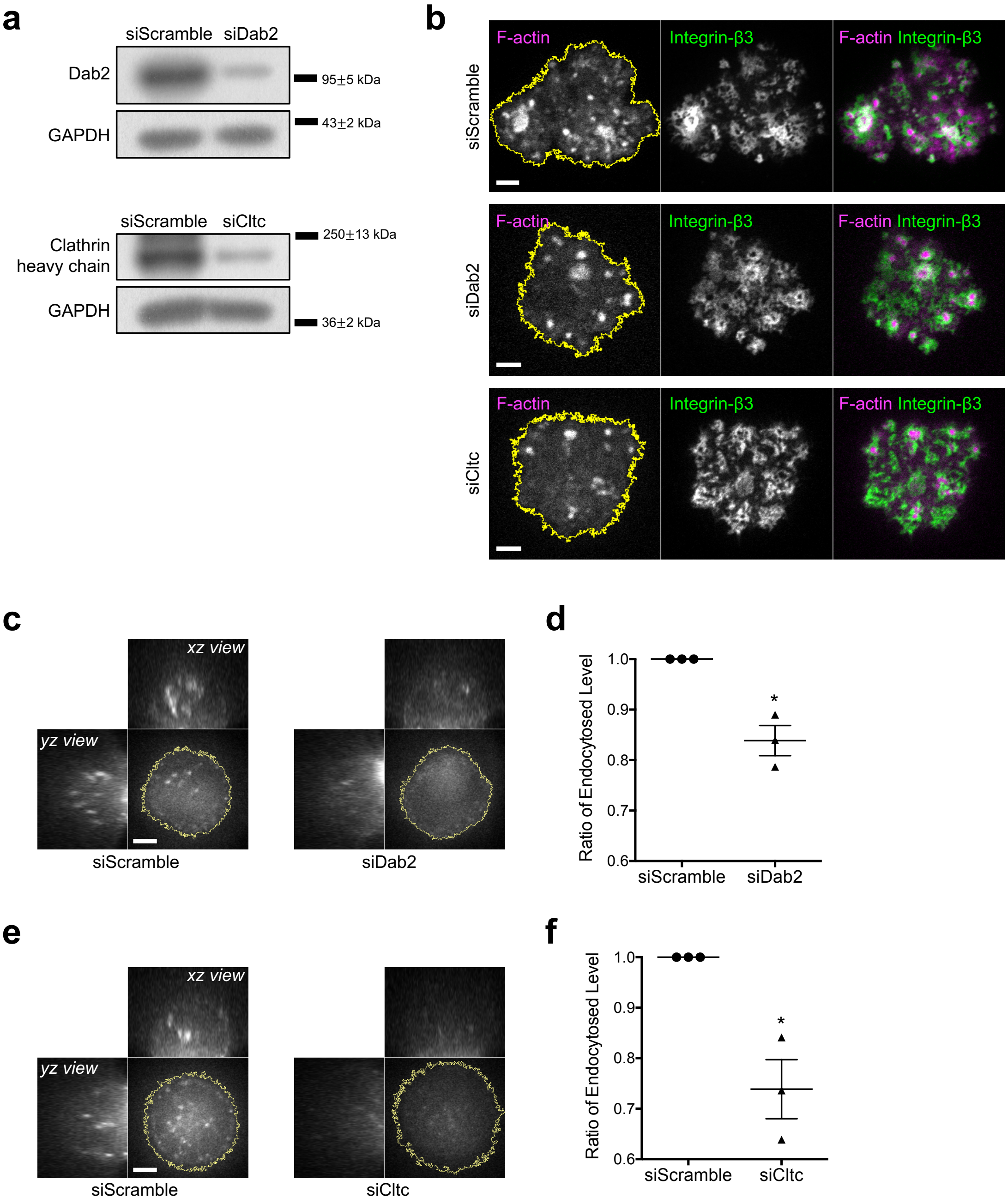

Supplementary Figure 3. (A) Western blots confirm the siRNA knockdowns of Dab2 and clathrin heavy chain (Cltc) in REF52 cell. (B) Knockdowns of Dab2 or Cltc does not impede the podosome formation. Podosomes are identified by integrin-beta3-GFP (podosome ring) and BPF2-UtrCH (F-actin marker, podosome core). (C-F) Knockdowns of Dab2 and Cltc individually causes the decrease of RGD-NA488 endocytosis level. Three-dimensional confocal images are shown with the z position from 2 μm to 20 μm (xz and yz view, 500nm z-step), while the image shown in xy view is at the z position of 5 μm above

the adhesion plane. Statistical information is in Supplementary Fig. 10H. Scale bars represent 5 μm. Error estimates are S.E.M.

Supplementary Figure 4

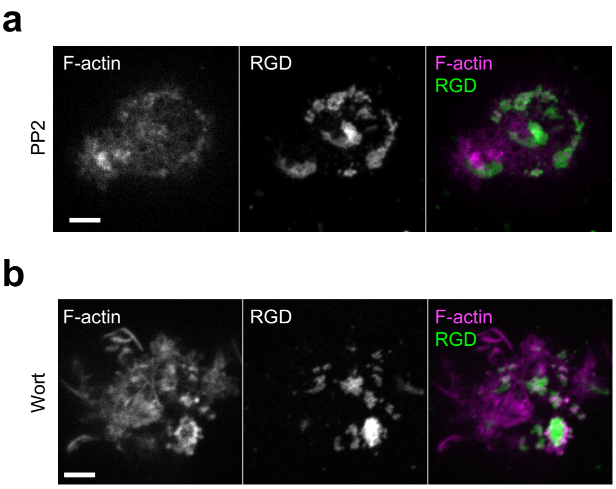

Supplementary Figure 4. Podosome formation is suppressed in PP2-treated (A) and wortmannin-treated (B) REF52 cells. Prominent assemblies of F-actin surrounded by RGD-NA680 are not observed. F-actin is labeled by BFP2-UtrCH in REF52 cell. Scale bars represent 5 μm.

Supplementary Figure 5

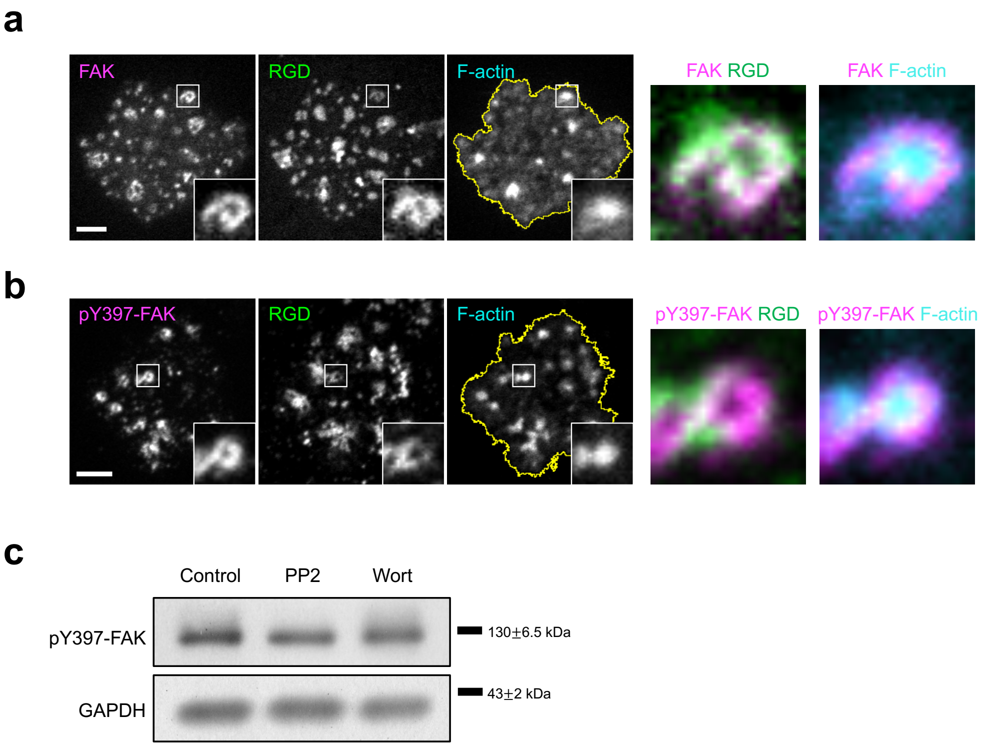

Supplementary Figure 5. (A-B) GFP-FAK and pY397-FAK localize at RGD-NA680 labeled podosome ring and surround the F-actin of podosome core. F-actin is labeled by BFP2-UtrCH in REF52 cell. (C) Western blot confirms that the treatments of PP2 and wortmannin do not suppress the combined pY397-FAK levels. REF52 cells are not detached from the substrate before collecting cell lysate for western blot. Scale bars represent 5 μm.

Supplementary Figure 6

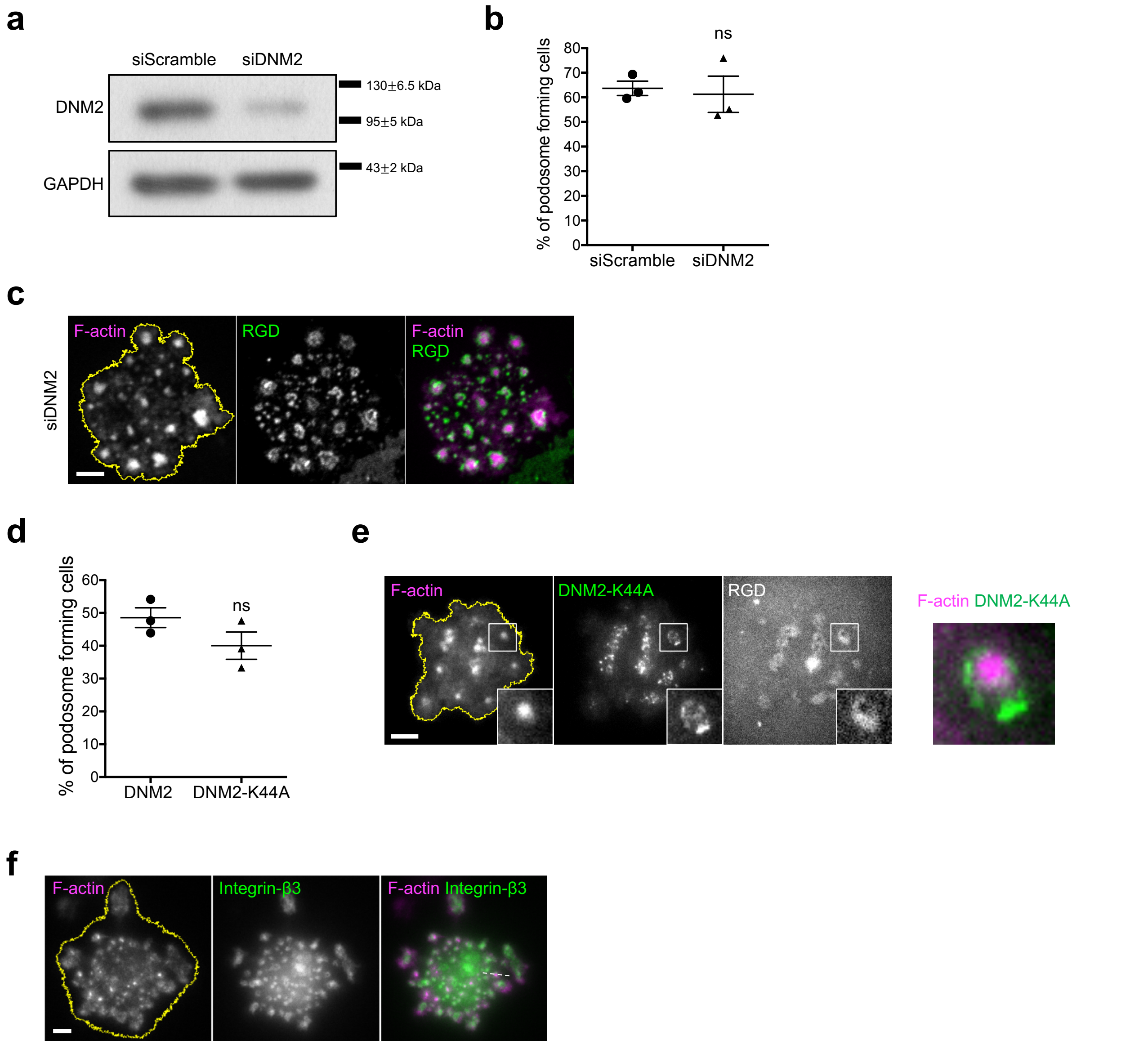

Supplementary Figure 6. (A) Western blot confirms the siRNA knockdown of DNM2 in REF52 cell. (B-C) Knockdown of DNM2 does not significantly alter the percentage of podosome-forming cells on RGD-membrane. Podosomes are identified by CF594-phalloidin stained F-actin (podosome core) and RGD-NA680 (podosome ring). Statistical information is in Supplementary Fig. 10I. (D-E) The presence of DNM2-K44A-GFP does not significantly reduce the percentage of podosome-forming cells on RGD-membrane. DNM2-K44A-GFP localizes at the RGD-NA680 labeled podosome ring and surrounds the F-actin of podosome core (labeled by BPF2-UtrCH). Inset: the boxed region (5x5 μm^2).

Statistical information is in Supplementary Fig. 10J. (F) The ROI (dashed line) to generate the kymograph in Figure 4C. Scale bars represent 5 µm. Error estimates are S.E.M.

Supplementary Figure 7

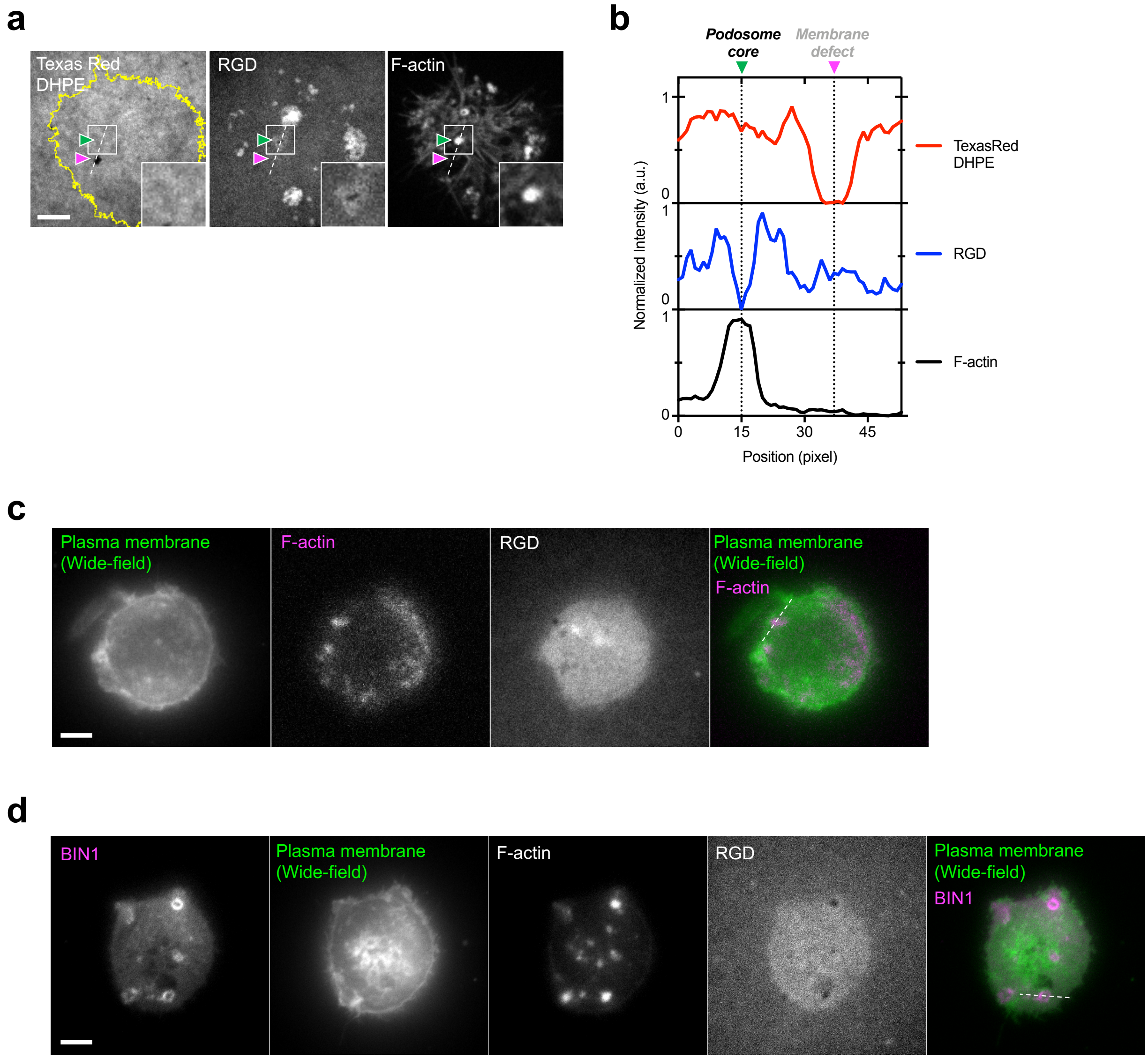

Supplementary Figure 7. (A) Texas Red DHPE labeled supported lipid membrane remains uniform at the podosome core when REF52 cells adhere on RGD-membrane. F-actin is labeled by BFP2-UtrCH. Inset: the boxed region (5x5 μm^2). (B) Line scan intensity profiles of Texas Red DHPE, RGD-NA680, and F-actin across a podosome core (green arrowhead) and a membrane defect (magenta arrowhead, lack of Texas Red signals) in (A). While F-actin of the podosome core localizes at the region of RGD depletion, the underlying supported lipid membrane remains intact and unpenetrated. (C-D) ROIs (dashed lines) to generate kymographs in Figure 5C and 5E, respectively. Scale bars represent 5 μm.

Supplementary Figure 8

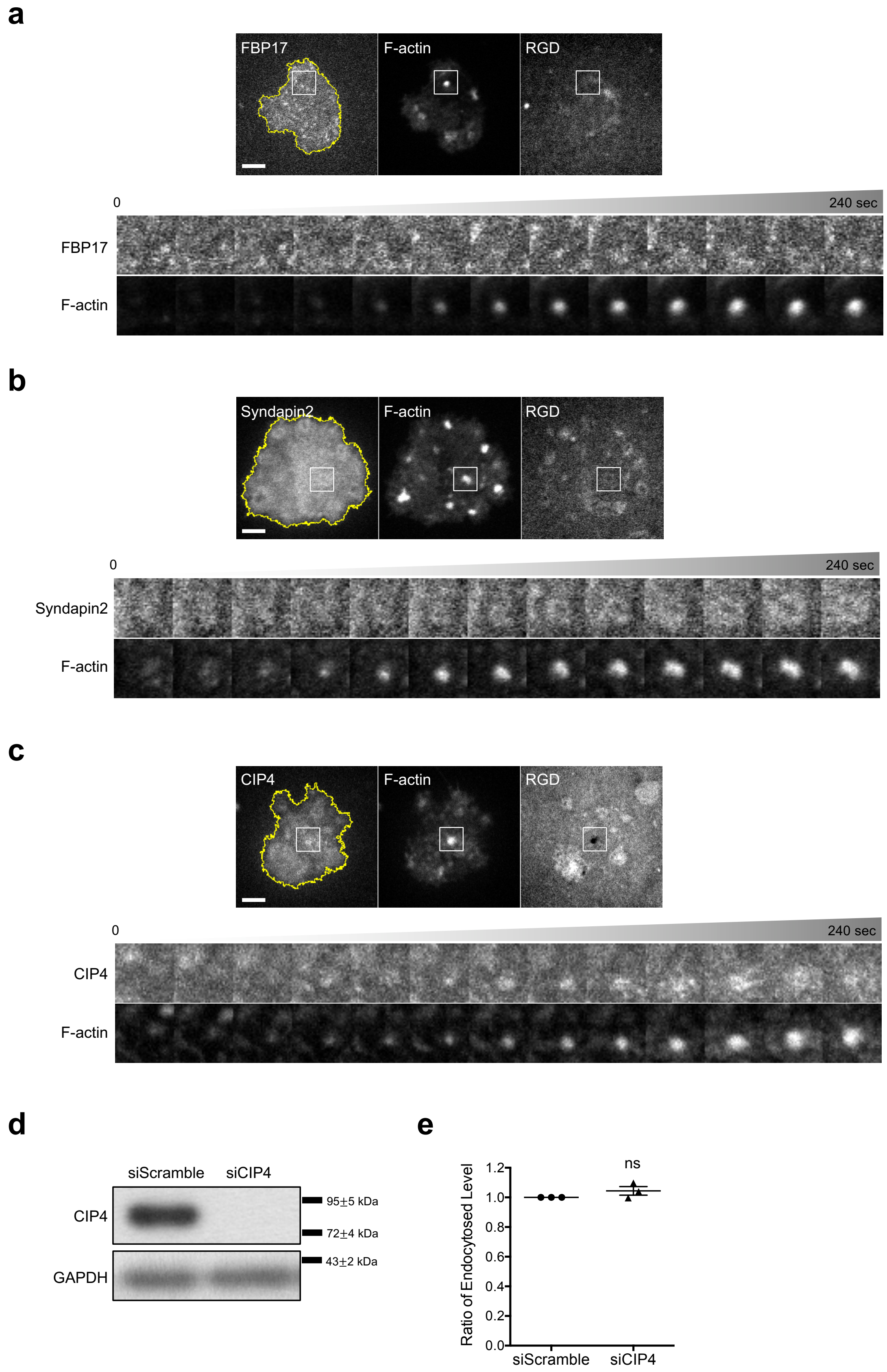

81

82 **Supplementary Figure 8.** (A-C) mCherry-FBP17 and mCherry-syndapin2 are not

83 enriched at the podosome. F-BAR domain protein mCherry-CIP4 is recruited to the

podosome core and localizes at dot-like assembly of F-actin. F-actin is labeled by BPF2-UtrCH in REF52 cell. Insets: the boxed region (5x5 μm^2). (D) Western blot confirms the siRNA knockdown of CIP4. (E) Knockdown of CIP4 does not cause significant changes in RGD-NA488 endocytosis level. Statistical information is in Supplementary Fig. 10K. Scale bars represent 5 μm. Error estimates are S.E.M.

Supplementary Figure 9

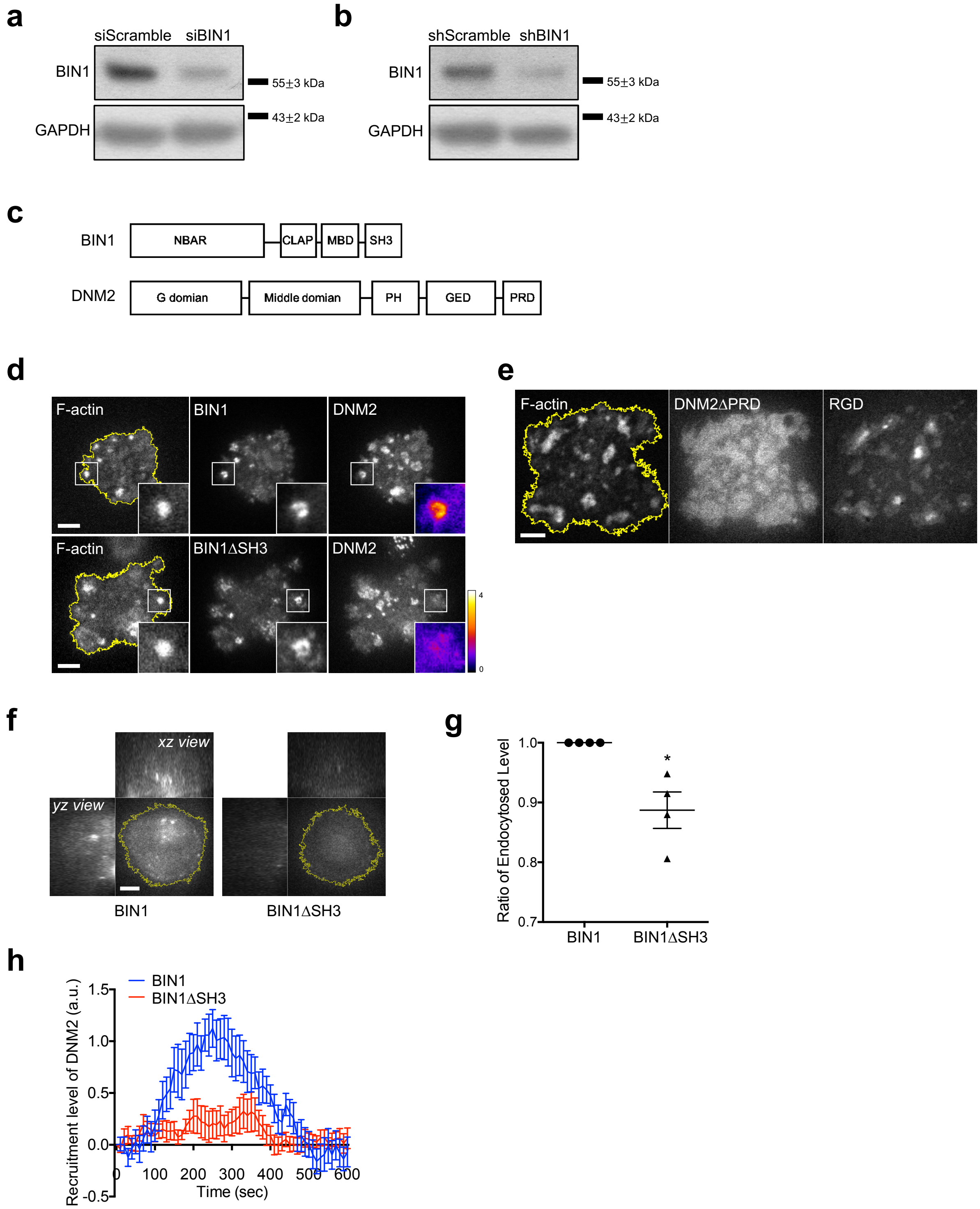

Supplementary Figure 9. (A-B) Western blots confirm the knockdown of BIN1 in REF52 cell by siRNA and shRNA, respectively. (C) Schematic diagrams of domain and motif of BIN1 and DNM2. (D) Both BIN1-mCherry and BIN1ΔSH3-mRuby2 (aa 1-404) are enriched at the podosome ring and surround the F-actin of podosome core. Overexpression of

BIN1ΔSH3 suppresses the recruitment of DNM2-GFP to the podosome ring. F-actin is labeled by BPF2-UtrCH. Inset: the boxed region (5x5 μm^2). Ratiometric insets of DNM2 indicate the recruitment level. (E) DNM2ΔPRD-GFP is mostly cytosolic and is not enriched around the F-actin of podosome core (labeled by BPF2-UtrCH). (F-G) Overexpression of BIN1ΔSH3 suppresses RGD-NA488 endocytosis. Three-dimensional confocal images are shown with the z position from 2 to 20 μm (xz and yz view, 500nm z-step), while the image shown in xy view is at the z position of 5 μm above the adhesion plane. Statistical information is in Supplementary Fig. 10L. (H) Recruitment level of DNM2-GFP to the podosome ring is reduced when BIN1ΔSH3 is overexpressed. All intensity traces are synchronized by realigning F-actin peak intensity at 400-sec. BIN1 samples are analyzed from 14 podosomes from 5 cells in 3 independent experiments. BIN1ΔSH3 samples are analyzed from 11 podosomes from 4 cells in 3 independent experiments. Scale bars represent 5 μm. Error estimates are S.E.M.

Supplementary Figure 10

a

Protein	No. of positive podosome	No. of negative podosome	Total podosome reviewed	Ratio of positive podosome
Dab2	130	1	131	0.9924
DNM2	99	2	101	0.9802
BIN1	101	0	101	1

b

Protein	Total No. of RGD Puncta	No. of Rab-positive RGD	Ratio of Rab-positive RGD
Rab5	219	86	0.3927
Rab7	177	93	0.5254
Rab11	241	65	0.2967
Rab4	243	137	0.5638

c

	Name	Mean	SEM	P value	n	N
Fig. 2A	Control	43.62	2.425	N.A.	332	4
	PP2	25.07	3.65	0.0075	336	4
Fig. 2D	Control	44.01	3.755	N.A.	231	3
	Wortmannin	24.35	2.719	0.0161	255	3

d

	Name	Mean	SEM	P value	n	N
Fig. 2C	Control	1	0	N.A.	332	4
	PP2	0.8092	0.02607	0.0053	336	4
Fig. 2F	Control	1	0	N.A.	231	3
	Wortmannin	0.8181	0.0204	0.0123	255	3

e

	Name	Mean	SEM	P value	n	N
Fig. 3B	siScramble	1	0	N.A.	369	4
	siDNM2	0.8061	0.03853	0.0151	256	4

f

	Name	Mean	SEM	P value	n	N
Fig. 3D	DNM2	1	0	N.A.	264	3
	DNM2-K44A	0.8472	0.02564	0.027	232	3

g

	Name	Mean	SEM	P value	n	N
Fig. 6E	siScramble	1	0	N.A.	517	5
	siBIN1	0.8398	0.04304	0.0204	558	5

h

	Name	Mean	SEM	P value	n	N
	siScramble	1	0	N.A.	239	3
Fig. S3D	siDab2	0.8387	0.02979	0.0325	219	3
Fig. S3F	siCltc	0.7387	0.05853	0.0467	197	3

i

	Name	Mean	SEM	P value	n	N
Fig. S6B	siScramble	63.67	2.914	N.A.	282	3
	siDNM2	61.24	7.374	0.7821	177	3

j

	Name	Mean	SEM	P value	n	N
Fig. S6D	DNM2	48.56	3.002	N.A.	238	3
	DNM2-K44A	40.04	4.149	0.1778	236	3

k

	Name	Mean	SEM	P value	n	N
Fig. S8E	siScramble	1	0	N.A.	246	3
	siCIP4	1.044	0.02906	0.2675	230	3

l

	Name	Mean	SEM	P value	n	N
Fig. S9G	BIN1	1	0	N.A.	338	4
	BIN1ΔSH3	0.8871	0.03042	0.034	322	4

Supplementary Figure 10. (A) Percentages of podosomes that are positive in Dab2, DNM2, and BIN1 in REF52 cells. (B) Percentages of Rab5, Rab7, Rab11, and Rab4-positive RGD puncta. (C-L) The statistical information of the specified figure. “n” represents the number of cells quantified, while “N” represents the number of experiments performed.

Supplementary Figure 11

a

Figure 2I and 2J

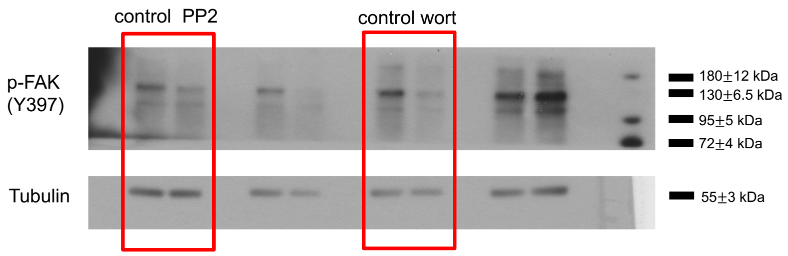

b

Supplementary Figure 3A

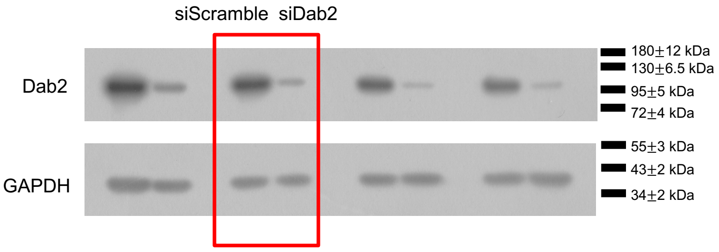

c

Supplementary Figure 3A

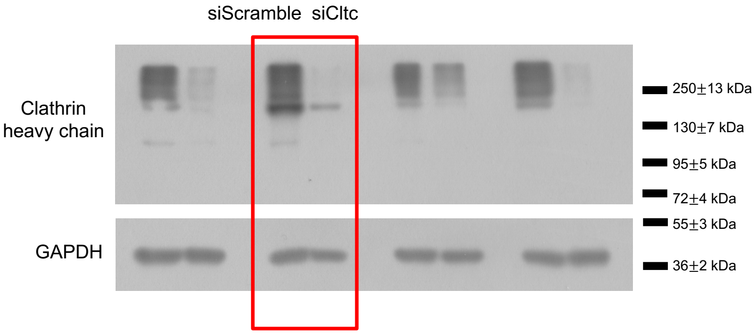

d

Supplementary Figure 5C

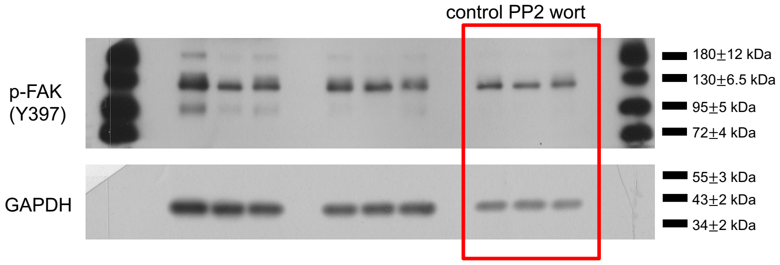

Supplementary Figure 11 (continued)

e

Supplementary Figure 6A

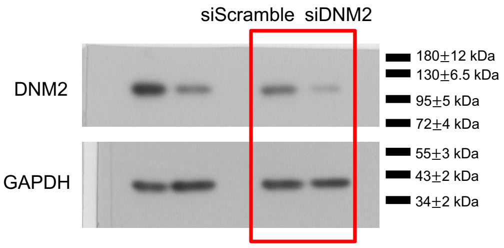

f

Supplementary Figure 8D

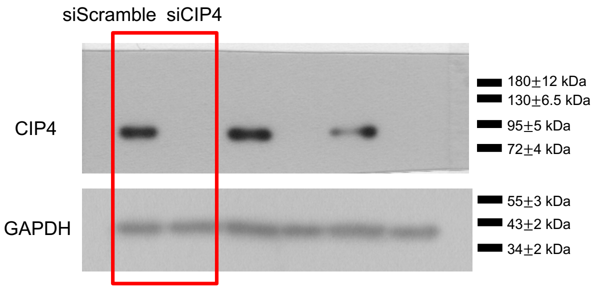

g

Supplementary Figure 9A and 9B

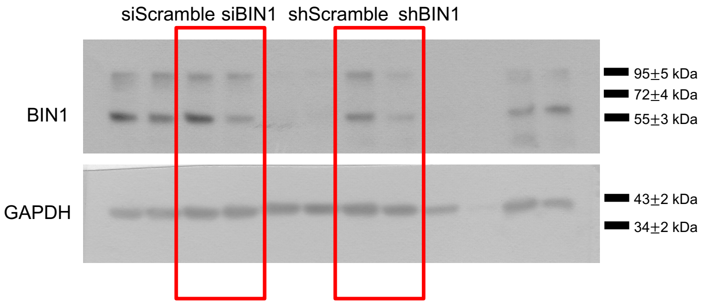

Supplementary Figure 11. (A-G) Full western blots of the specified figure.

Supplementary Table 1

**Name**	**Sequence**
siBIN1 (SR511599)	AAA GAG AUG AGU AAG CUC AAU CAGA
shBIN1 (reverse transcription of siBIN1)	AAA GAG ATG AGT AAG CTC AAT CAGA
siDNM2 (SR501378)	GAG AUC AGC UAC GCC AUU AAG AACA
siDab2 (SR505444)	CUC AGC UAA UUG CAU CAU UUA CCAA
siCltc (SR507164)	AGG UCA AUU CUC UAC UGA UGA GCTT
siCIP4 (SR514456)	GUU GUC AUU UCA GAC CUU UCU CCCT
