# Supplementary material for: Podosome formation promotes plasma membrane invagination and integrin-β3 endocytosis on a viscous RGD-membrane
Source: Commun Biol. 2020 Mar 13;3:117. doi: 10.1038/s42003-020-0843-2 (PMC7070051; doi:10.1038/s42003-020-0843-2)
Supplement: Supplementary file 2 — Description of Additional Supplementary Files [file 42003_2020_843_MOESM2_ESM.pdf]

Description of Additional Supplementary Items

Cao et al., Podosome formation promotes plasma membrane invagination and integrin-β3 endocytosis on a viscous RGD-membrane.

1. Supplementary Data 1

All source data underlying the graphs and charts of this work.

2. Supplementary Movie Legend

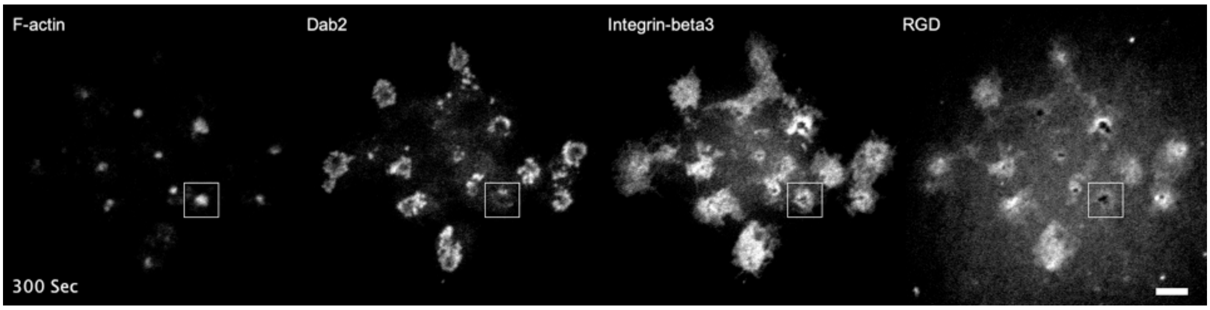

Supplementary Movie 1. Podosome formation of REF52 cell on RGD-membrane. F-actin, visualized by BFP2-UtrCH polymerizes and forms the podosome core. RGD-NA680 and integrin-β3-GFP clusters reorganize into doughnut-shaped adhesion and form the podosome ring. mCherry-Dab2 localizes at integrin-β3-GFP of the podosome ring and dissociates during podosome formation (e.g. the boxed region). Time interval 10 sec. Scale bar 5 μm.

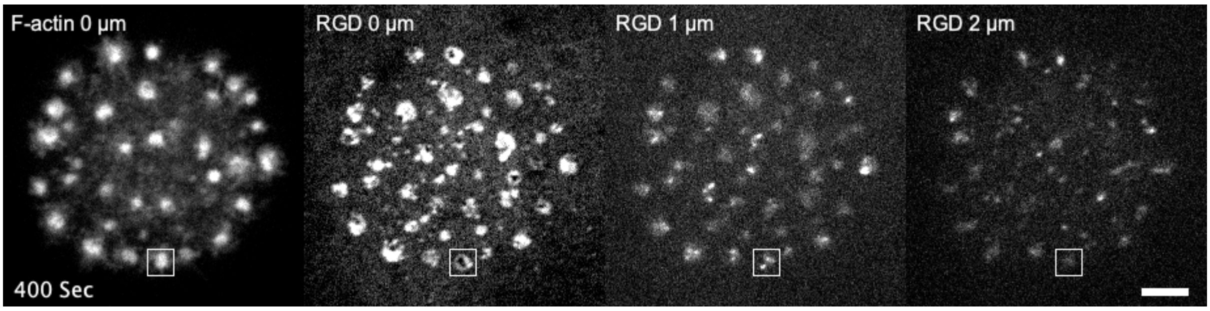

Supplementary Movie 2. RGD-NACB are internalized during the podosome formation. As F-actin polymerizes, the intensity of RGD-NACB at the adhesion decreases, while those at 1 μm and 2 μm above the adhesion plane increase (e.g.

the boxed region). F-actin is labeled by BPF2-UtrCH in REF52 cell. Time interval 20 sec. Scale bar 5 µm.

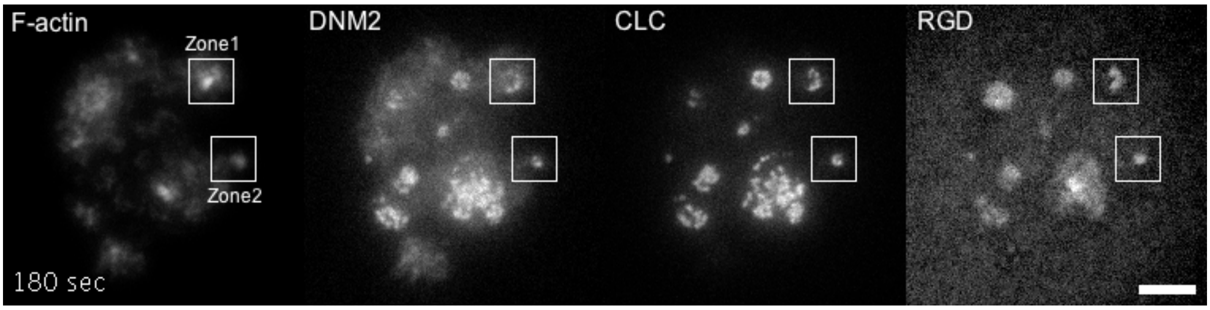

Supplementary Movie 3. During the podosome formation, DNM2-mCherry becomes enriched at the podosome ring, while mTagBFP2-CLC gradually dissociates (e.g. zone 1; with prominent dot-like F-actin polymerization). In a non-podosome RGD-NA680 cluster, the levels of DMN-mCherry and mTagBFP2-CLC remain unchanged (e.g. zone 2; without prominent F-actin polymerization). F-actin is labeled by BPF2-UtrCH in REF52 cell. Time interval 10 sec. Scale bar 5 µm.

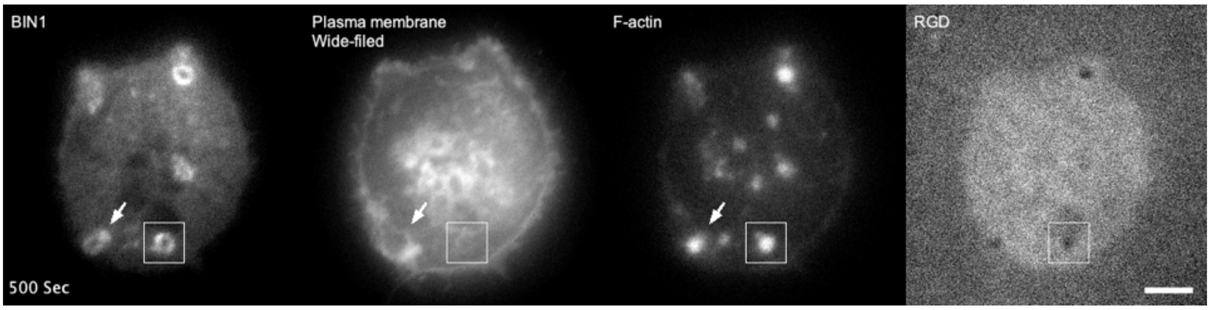

Supplementary Movie 4. The enrichment of BIN1-mCherry and plasma membrane invagination (visualized by PM-GFP) are synchronized as F-actin polymerizes at the podosome (e.g. the boxed region and arrow). F-actin is labeled by BPF2-UtrCH in MEF cell. Time interval 10 sec. Scale bar 5 µm.

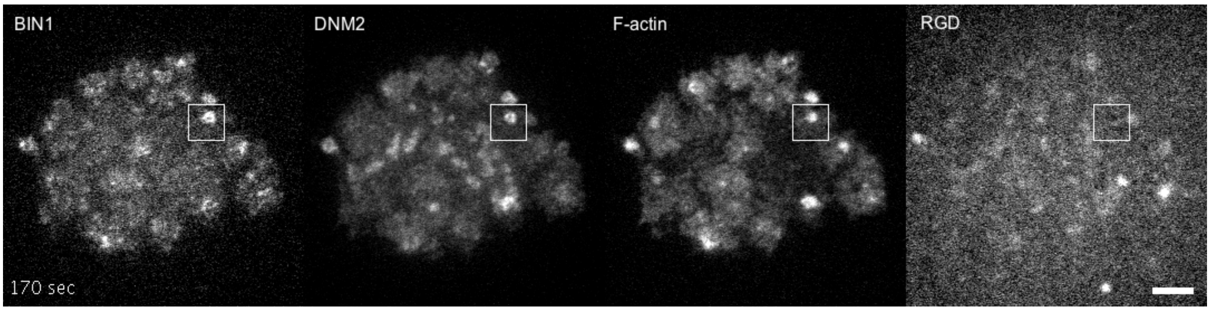

Supplementary Movie 5. BIN1-mCherry colocalizes with DNM2-GFP and surrounds the F-actin of podosome core. The recruitment of BIN1 is specific to podosome adhesions (e.g. the boxed region) and is missing at non-podosome adhesions. F-actin is labeled by BPF2-UtrCH in REF52 cell. Time interval 2 sec. Scale bar 5 µm.

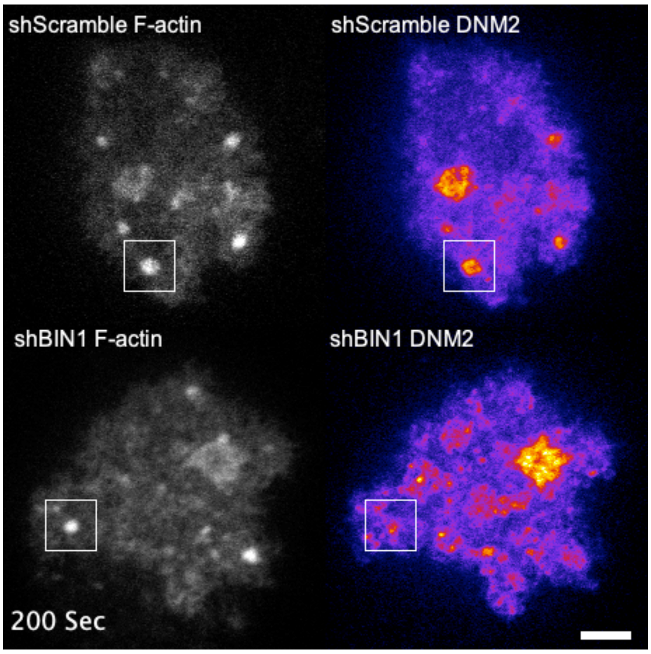

Supplementary Movie 6. Knockdown of BIN1 results in the decrease of DNM2-GFP recruitment at the podosome ring (e.g. the boxed region). Ratiometric images of DNM2 indicate the recruitment level, and the pseudo-color intensity scale is in Figure 6F. F-actin is labeled by BPF2-UtrCH in REF52 cell. Time interval 10 sec. Scale bar 5 µm.
