# Supplementary material for: Podosome formation promotes plasma membrane invagination and integrin-β3 endocytosis on a viscous RGD-membrane
Source: Commun Biol. 2020 Mar 13;3:117. doi: 10.1038/s42003-020-0843-2 (PMC7070051; doi:10.1038/s42003-020-0843-2)
Supplement: Supplementary file 10 — Reporting Summary [file 42003_2020_843_MOESM10_ESM.pdf]

nature research

Corresponding author(s): Cheng-han Yu

Last updated by author(s): Feb 8, 2020

Reporting Summary

Nature Research wishes to improve the reproducibility of the work that we publish. This form provides structure for consistency and transparency in reporting. For further information on Nature Research policies, see Authors & Referees and the Editorial Policy Checklist.

Statistics

For all statistical analyses, confirm that the following items are present in the figure legend, table legend, main text, or Methods section.

n/a	Confirmed	
☐	☒	The exact sample size (*n*) for each experimental group/condition, given as a discrete number and unit of measurement
☐	☒	A statement on whether measurements were taken from distinct samples or whether the same sample was measured repeatedly
☐	☒	The statistical test(s) used AND whether they are one- or two-sided<br>*Only common tests should be described solely by name; describe more complex techniques in the Methods section.*
☐	☒	A description of all covariates tested
☒	☐	A description of any assumptions or corrections, such as tests of normality and adjustment for multiple comparisons
☒	☐	A full description of the statistical parameters including central tendency (e.g. means) or other basic estimates (e.g. regression coefficient) AND variation (e.g. standard deviation) or associated estimates of uncertainty (e.g. confidence intervals)
☒	☐	For null hypothesis testing, the test statistic (e.g. *F*, *t*, *r*) with confidence intervals, effect sizes, degrees of freedom and *P* value noted<br>*Give P values as exact values whenever suitable.*
☒	☐	For Bayesian analysis, information on the choice of priors and Markov chain Monte Carlo settings
☒	☐	For hierarchical and complex designs, identification of the appropriate level for tests and full reporting of outcomes
☒	☐	Estimates of effect sizes (e.g. Cohen's *d*, Pearson's *r*), indicating how they were calculated

Our web collection on statistics for biologists contains articles on many of the points above.

Software and code

Policy information about availability of computer code

Data collection	Metamorph and Volocity were used to control and collect the images from the microscopes.
Data analysis	ImageJ, Imaris, and volocity were used to quantify the images.

For manuscripts utilizing custom algorithms or software that are central to the research but not yet described in published literature, software must be made available to editors/reviewers. We strongly encourage code deposition in a community repository (e.g. GitHub). See the Nature Research guidelines for submitting code & software for further information.

Data

Policy information about availability of data

All manuscripts must include a data availability statement. This statement should provide the following information, where applicable:

- Accession codes, unique identifiers, or web links for publicly available datasets
- A list of figures that have associated raw data
- A description of any restrictions on data availability

Data available on request from the authors.

Field-specific reporting

Please select the one below that is the best fit for your research. If you are not sure, read the appropriate sections before making your selection.

☒ Life sciences ☐ Behavioural & social sciences ☐ Ecological, evolutionary & environmental sciences

For a reference copy of the document with all sections, see nature.com/documents/nr-reporting-summary-flat.pdf

Life sciences study design

All studies must disclose on these points even when the disclosure is negative.

Sample size	N/A
Data exclusions	N/A
Replication	Biological repeats were performed more than 3 times in each experiment.
Randomization	N/A
Blinding	N/A

Reporting for specific materials, systems and methods

We require information from authors about some types of materials, experimental systems and methods used in many studies. Here, indicate whether each material, system or method listed is relevant to your study. If you are not sure if a list item applies to your research, read the appropriate section before selecting a response.

Materials & experimental systems

n/a	Involved in the study	
☐	☒	Antibodies
☐	☒	Eukaryotic cell lines
☒	☐	Palaeontology
☒	☐	Animals and other organisms
☒	☐	Human research participants
☒	☐	Clinical data

Methods

n/a	Involved in the study	
☒	☐	ChIP-seq
☒	☐	Flow cytometry
☒	☐	MRI-based neuroimaging

Antibodies

Antibodies used	Immunofluorescent staining: Primary antibodies included activated human integrin-β3 antibody (1:25, anti-LIBS2 epitope clone ab62, Millipore MABT27) and pFAK-Y397 (1:50, Thermo Fisher Scientific 44-624G). Secondary antibodies included AF594-anti-mouse (1:1000, Thermo Fisher Scientific A-21203) and AF594-anti-rabbit (1:1000, Thermo Fisher Scientific A-21207). Western blotting: Primary antibodies included pFAK-Y397 (1:1000, Thermo Fisher Scientific 44-624G), anti-BIN1 clone 99D (1:1500, Millipore 05-449), anti-DNM2 clone 27 (1:1000, BD Biosciences 610263), anti-Dab2 (D7O9T) (1:1000, Cell Signaling Technology 12906), anti-Clathrin heavy chain clone 23 (1:1000, BD Biosciences 610499), anti-CIP4 clone 21 (1:1000, BD Biosciences 612557), anti-tubulin alpha (G436) (1:10000, Bioworld BS1699) and anti-GAPDH (1:10000, Thermo Fisher Scientific AM4300). Secondary antibodies included anti-mouse HRP (1:2000, Santa Cruz sc-516102) and anti-rabbit HRP (1:2000, Cell Signalling Technology 7074).
Validation	Based on manufacturer's instructions.

Eukaryotic cell lines

Policy information about cell lines

Cell line source(s)	Rat embryonic fibroblast (REF52) was a gift from Dr. Benjamin Geiger, Weizmann Institute of Science, Rehovot, Israel. RPTPα +/+ mouse embryonic fibroblast (MEF) was a gift from Dr. Sap Jan, New York University School of Medicine.
Authentication	Not authenticated
Mycoplasma contamination	All cell lines were negative in mycoplasma contamination.
Commonly misidentified lines (See ICLAC register)	N/A
